# Which construal level combinations generate the most effective interventions? A field experiment on energy conservation

**DOI:** 10.1371/journal.pone.0209469

**Published:** 2019-01-17

**Authors:** Anouk M. Griffioen, Michel J. J. Handgraaf, Gerrit Antonides

**Affiliations:** Urban Economics, Social Sciences Group, Wageningen University, KN, Wageningen, The Netherlands; University College Cork National University of Ireland, IRELAND

## Abstract

Many campaigns targeting pro-environmental behavior combine multiple approaches without properly understanding how these different approaches interact. Here we study the effect of such combinations. We apply construal level theory to classify different intervention approaches, which can either be at a high construal level (abstract and distant) or at a low construal level (concrete and proximal). In a field experiment we recruited 197 students living in one-person apartments in an all-inclusive student housing facility. We objectively measured their individual electricity and warm water use, and measured psychological variables through surveys. We expected that the (commonly considered superior) combination between a high and a low construal level approach would be least effective. Participants were randomly assigned to a 2(Construal Level: low vs. high) × 2(Social Distance: low vs. high) plus control condition mixed-model design targeting a reduction in warm water use. Our findings suggest that a congruent combination at a high construal level (i.e., the high construal level condition combined with the high social distance condition) has the largest effect on warm water use and that spillover to electricity use is most likely to occur when a high construal level is used (i.e., high social distance). Moreover, especially participants who valued nature and the environment less were most strongly influenced by the combination of two high construal level approaches. In sum, our study suggests that when designing interventions one should consider the construal level and when targeting pro-environmental behavior high construal levels appear most appropriate.

## Introduction

Environmental campaigns frequently appeal to several motivations at the same time in an attempt to achieve maximum impact on behavior. Policy makers, for example, use slogans such as “Save Money, Save Energy, Save the Environment” [1], which is expected to be effective based on the premise that some people value the environment, whereas others value the financial benefits of saving energy [2]. Although some studies support this premise [3–5], Schwartz et al. [6] showed the contrary; appealing to both financial and environmental benefits of an energy saving program at the same time was less effective than appealing to the environmental benefits alone. We argue that this may be due to the construal level (i.e., the level of abstraction) these appeals elicit among consumers. In this example, the monetary benefits of energy saving behavior are more concrete, personally relevant and will likely materialize in the near future, which is associated with a low construal level. In contrast, the environmental benefits of energy saving behavior are far more abstract, less personally relevant and the consequences are uncertain, which corresponds to a high construal level. We argue that two appeals that are at different construal levels are not very effective when targeting pro-environmental behavior. We, therefore, aim to test whether construal level is a possible explanation for the (in)effectiveness of particular combinations of interventions.

To date, it remains largely unclear how to combine different interventions in an effective manner [7,8]. Notwithstanding the fact that some combinations do not work well together, combinations in general do have the potential of having a larger impact on behavior as compared to single approaches. The aim of our current research is to study the effects of combinations of interventions at different construal levels on pro-environmental behavior, and specifically on energy conservation behavior. Besides studying the effects on targeted behavior, we also investigate effects on other, related (spillover) behavior. By manipulating the construal level of two interventions we aim to understand whether combinations that are at the same level or at different levels of construal are more effective when targeting energy saving behavior. We specifically study the effects on curtailment (i.e., habitual energy use) behavior, and how changes in such day-to-day behavior affect overall energy use. We test the effectiveness of these combinations in a unique field setting where we can, besides self-report measures, objectively measure individual energy and water use over the course of our experiment.

### Construal level theory

As people can only experience the here and now, construal level theory poses that one has to imagine events that are not taking place right here and now at some level of abstraction [9]. This indicates that people can think of an event at either a low level of abstraction, which means the event is construed in a very concrete manner, or at a high level, where the event is construed more abstractly. For example, when one decides to recycle a plastic bottle, at a low construal level one looks for the bin and throws it in, whereas at a high construal level one may think of the consequences of plastic garbage for the environment [10]. Construal level theory has been used as an overarching framework to understand behavioral tendencies, including decision-making, behavior in organizational contexts [11], and interpersonal processes [12].

Construal level theory indicates that when people think at a low construal level they think more about the context-specific features of behavior and people are less able to separate important goals from other unimportant features [13]. Therefore, when people think at a low construal level, they are more susceptible to the influence of contextual factors that may either promote or inhibit pro-environmental behavior, and people are concerned with “how” they can perform certain behavior [9,14]. At a high construal level, thoughts tend to become more coherent and structured, and people often leave out irrelevant details [15]. In behavioral terms, a high construal level suggests that behavior is mostly guided by inner values and superordinate goals, and that people are concerned about “why” they engage in certain behavior [16].

High and low construal level thinking are both used naturally by people when thinking about objects or behavior [17]. Besides the fact that people can spontaneously shift between high and low levels of construal, different construal levels can also be experimentally manipulated by message framing or offering contexts that influence the construal level [18]. Moreover, construal level can be indirectly manipulated by varying one of the psychological distance dimensions, which include temporal, social, spatial and hypothetical distance [13,15]. Construal level theory poses that the more psychologically distant an object, event or behavior is, the more abstractly it is perceived by people, thus implying a higher construal level. In terms of interventions with a focus on environmental benefits, this is associated with a large psychological distance on all four dimensions [19,20], whereas personal benefits (e.g., financial) relate to smaller psychological distance on those dimensions [21,22]. For example, when environmental benefits of saving energy are highlighted, the exact consequences of this behavior concern benefits to society at large (social) and the entire planet (spatial), will only materialize later in time (temporal), and, finally, will be quite uncertain (hypothetical). Even though environmental problems could be framed as being more psychologically proximal, one inherent feature of environmental problems is that people have the tendency to believe that the consequences are more likely to affect other people than themselves [23]. Therefore, the social distance of environmental problems is often experienced as being large and appealing to other benefits that are closer in social distance may be the only way to decrease the distance on this dimension.

### Combining construal levels: Congruent versus incongruent interventions

Interventions frequently combine multiple approaches, based on the somewhat simplistic reasoning that combinations have the potential of having a larger effect across more individuals. From a construal level theory perspective, there are two ways in which combinations could be formed: either the two (or more) interventions are at different construal levels (high and low), or the interventions are at the same construal level (high and high, or low and low).

Some studies have looked at the potential benefits of two manipulations that are at different construal levels. Most studies show that combining high and low construal level interventions are not very effective [6,24], with the exception of one study that found that the combination of a high and low construal level increased participants’ willingness to donate to charity [25]. Construal level theory provides a possible explanation for the ineffectiveness of combinations that are at different construal levels, based on the premise that people attend and process information differently depending on their construal level. According to construal level theory, when a high and a low construal level approach are combined, people may give more attention to the low construal level factor, since they can relate more easily to the concrete, low level component as it represents “[t]he pushes and pulls of everyday life” (p. 92; 10). This suggests that when high and low construal levels are combined, the effect of the high construal level approach is completely cancelled out by the low construal level component.

Here, we argue that people process information more efficiently when two intervention approaches are at the same level of construal. In line with this idea, in a series of lab studies, Amit, Algom, and Trope [26] found that when there was fit between the presentation medium (words vs. pictures) and psychological distance, processing was more efficient in recall experiments and in a response time experiment. In relation to pro-environmental behavior, previous studies found that a match in terms of construal level–either at a high or low level–was more effective in promoting pro-environmental intentions and behavior than a mismatch [24,27]. Moreover, studies that aim to stimulate pro-environmental behavior seem to favor appeals to self-interest and pro-social motivations, either at the same time or separately. Goldsmith, Newman, and Dhar [28] showed that congruent combinations were again most effective. Congruent combinations were operationalized as the combination between low construal level thinking and self-interest appeals, and between high construal level thinking and pro-social appeals. We extend this logic and argue that appeals to either self-interest or pro-social benefits of pro-environmental behavior differ on at least one of the psychological distance dimensions: social distance. Specifically, appeals to self-interest are small in social distance and appeals to pro-social motivations can be seen as large in social distance. This reasoning is in line with previous work, showing that self-interest appeals evoke low construal level thinking [29]. As such, we argue that the combinations were effective because they were aligned in terms of their (indirect) construal levels. Previous research thus supports the idea that a fit in construal level of different interventions plays an important role in how people attend to information and how this influences their decision making and behavior. Therefore, in this paper we argue that congruent construal level combinations will be more effective in changing behavior than incongruent combinations.

### Congruent construal level manipulations: High versus low

If indeed construal level combinations that are at the same level are the most effective, the question remains whether low or high construal level combinations work best. Clearly, high and low level construals are processed differently, which implies that different factors drive the ultimate decisions and behavior. In terms of pro-environmental behavior, some researchers have argued in favor of high construal levels [10], whereas other researchers have argued that environmental problems should be communicated as a present and personal risk and thus at a lower construal level [30,31]. Important to note is that people may arrive at the exact same decision via either the high or the low construal level path [32]. Therefore, it is possible that high and low construal level approaches are equally effective as long as combinations are congruent in terms of construal level.

In terms of decision making processes, at a low construal level people are mostly directed by the concrete, detail-specific features of events [14]. Moreover, low level of construal approaches have been associated with feasibility concerns [33], suggesting that when a behavior is easy and feasible people will most likely engage in that behavior. However, when the situation does not facilitate the behavior, it is more likely that people will focus on the potential barriers or extra effort they have to exert and thus might not change their behavior.

For high construal level approaches, people focus more on the general goal the behavior is serving [14], which may be beneficial in the case of pro-environmental behavior. Goldsmith, Newman, and Dhar [28] found that high construal level appeals are most effective when people were thinking in more abstract, high level terms when stimulating green product choice. Additionally, when people think at a high construal level they are usually guided by their inner values [29]. Making people act upon their inner values can be potentially beneficial when trying to stimulate pro-environmental behavior, as people often do not act upon their inner values in many everyday situations [14]. However, as high construal levels make people act more upon their values, this may only be beneficial when people actually value the environment [34] and the high level of construal approach specifically highlights existing pro-environmental values. We expect that both high and low construal level approaches can be effective, but that behavior is guided by different motivations or considerations.

### The role of biospheric values

When people think at a high level of construal, pre-existing values seem to be an important determinant for their decisions and behavior [9,16]. In terms of pro-environmental behavior, previous studies show that especially biospheric values have a strong positive relation with pro-environmental intentions and behavior [35,36]. In contrast, egoistic values have negative associations with pro-environmental behavior [37]. Therefore, people who strongly endorse biospheric values generally display more pro-environmental behavior, and high construal levels are expected to appeal to these inner values which may in turn lead to even more pro-environmental behavior. Indeed, Brügger et al. [38] show that when people think of climate change as a distant issue, they act more upon their (altruistic and) biospheric values. Likewise, Bolderdijk, Gorsira, Keizer, and Steg [39] showed that only people who strongly endorsed the environment were affected by a movie about environmental consequences of bottled water in terms of their policy support and intentions.

At the same time, however, when people do not strongly value the environment, but instead endorse egoistic values, high construal level approaches may lead to more egoistic behavior, as people act in line with these values [34]. This is rather unfortunate, as people who score higher on egoistic values may already engage less in pro-environmental behavior, whereas they have the biggest potential in terms of behavior change. This is in contrast to people who highly value the environment and already engage in many pro-environmental activities, which suggests that there is little room for improving their current behavior. In line with this reasoning, Schoenefeld and McCauley [40] found that people who scored higher on self-transcendent (i.e., altruistic and biospheric) values were not affected by information about climate change impacts, either at a high or low construal level. This indicates that people who value the environment may not be affected by manipulations targeting behavior change, for example, because they already act pro-environmentally. Although values are relatively stable traits that do not change in a short period of time [41], making particular values temporally salient can influence subsequent choices and behavior [42–44]. Therefore, one way to motivate individuals who have weaker biospheric values is by strengthening their biospheric values, which may lead to more pro-environmental behavior than before [45]. When interventions strengthen biospheric values and are at a higher construal level this combination may thus be most effective in realizing behavior change.

### Spillover behavior

Besides the direct effects of construal level on a target behavior, pro-environmental behavior is usually not just about making one choice, but about making many choices across many different contexts over longer periods of time. Truelove, Carrico, Weber, Raimi, and Vandenbergh [46] call this the “net environmental impact,” which transcends the effect on the target behavior, and also considers the longevity of these effects and the effects on related behaviors (spillover). Spillover occurs when an intervention also affects another behavior than the targeted behavior. Pro-environmental spillover is positive when it leads to additional pro-environmental behavior and negative when it leads to less pro-environmental behavior [47].

It has been suggested that high construal level thinking may lead to behavior change that is more lasting over time and less context specific [10], and thus to more positive spillover. Moreover, Mullen and Monin [48] argue that when people think at a high construal level they focus on their values and superordinate goals, which leads to consistency across different behavioral domains, and thus to positive spillover. In contrast, low construal level thinking induces a focus on concrete actions and consequences, which may lead to no spillover at all. In line with this, Evans et al. [42] found that a combination of a high and low construal level (viz., appealing to environmental and financial benefits) did not lead to positive spillover, whereas only appealing to a high level of construal (viz., the environmental benefits of pro-environmental behavior) did lead to positive spillover. Therefore, we argue that high construal level combinations are the most effective in terms of spillover behavior and net environmental impact in general.

### Present research

Based on the assumption that combined intervention approaches can be more effective in stimulating pro-environmental behavior than single interventions, this research aims to investigate which combinations of high and low construal level interventions are most effective. We will measure effectiveness of combined construal level manipulations on a target behavior, warm water use, as well as on related behaviors, such as electricity use. We have chosen warm water use, as it is the second most impactful behavior in terms of energy consumption in the household (after space heating/cooling) and accounts for approximately 18% of household energy use [49]. Moreover, in our experiment warm water use was much more explicitly under control of our participants as compared to space heating/cooling, which is mostly automatic. In our experiment participants received two manipulations that were intended to affect their construal level (see Method section). The first manipulation was a commonly used direct construal level manipulation (i.e., “how versus why” task; [18]) and the second manipulation was an indirect construal level manipulation, by manipulating social distance. We specifically chose to manipulate social distance for two main reasons: environmental problems are usually perceived as being socially distant and previous work often contrasts personal with non-personal benefits (i.e., values; [6,28]). To be able to test the effectiveness of high and low construal level approaches as clean as possible, we wanted to test this in a situation in which personal (low construal level) benefits of energy conservation behavior are naturally lacking. Therefore, we studied this in a field experiment, in which participants did not pay for the energy they used. In this particular situation, we expect that combinations that are at the same level of construal (i.e., that have construal level fit) have a greater impact on the target behavior (warm water use) as compared to combinations that are at different levels of construal. This leads to *Hypothesis 1*: *Combinations at the same construal level will have a greater effect on warm water use reduction as compared to non-aligned combinations*.

Moreover, in terms of expected spillover, we expect that the combination of two high construal level approaches will have the largest impact on related behaviors (e.g., electricity use), as compared to the other combinations. This leads to *Hypothesis 2*: *The congruent high construal level combination will have a greater effect on spillover behavior compared to the other construal level combinations*.

Finally, values play an important role in terms of construal level and pro-environmental behavior. Based on previous research, we expect that biospheric values can influence behavior in one of two ways, which leads to two competing hypotheses. First of all, it could be that people who score high on biospheric values act more in line with these values when they are in the high construal level conditions, as compared to people who score lower on biospheric values [16]. Secondly, as people who score high on biospheric values are also expected to already engage in more pro-environmental behavior, it could be that people who actually score lower on biospheric values are affected to a larger extent by high construal levels than people who score high on biospheric values.

## Methods

To test the effectiveness of the construal level combinations we ran an experimental field study. We focused on reducing warm water use at a student housing facility in the Netherlands. In this experiment, we manipulated construal level in two ways. The first manipulation, a direct construal level manipulation, was an adjusted version of the “how versus why” task by Freitas et al. [18], which has been applied in lab experiments, but has not been extensively tested in the field. Our second manipulation was an indirect construal level manipulation and influenced social distance by providing participants either with a gift to self (low social distance) or gift to other (a donation to a charitable organization; high social distance). This study has been approved by the Research/Assessment Committee from the Wageningen School of Social Sciences, Wageningen University. All participants provided written informed consent at the beginning of the study.

### Setting

In collaboration with an all-inclusive student housing facility, we installed detailed measurement equipment in 156 one-person apartments. This student housing facility provides high-end hotel-like rooms with private bathrooms. The installed equipment measures electricity use, warm water use, and presence in the room per participant on a minute-to-minute basis. This unique “living lab” setting, allows us to run experiments and collect and analyze objective behavioral data. Moreover, as each apartment is occupied by one person, we are able to directly link self-reported personal characteristics to the electricity and water use behavior in these rooms.

### Participants and design

Participants were randomly assigned to a 2 (Construal Level: low vs. high) × 2 (Social Distance: low vs. high) plus control condition in a between-subjects design. Participants were asked to fill out two surveys; one survey before the intervention as a baseline measurement, and one survey four weeks after the intervention. Participants (*n* = 197, *M*_*age*_ = 21.18, *SD*_*age*_ = 3.76, 53.3% female) were recruited in two waves. In the first wave of data collection in April 2015, 91 students (*M*_*age*_ = 22.13, *SD*_*age*_ = 3.98, 50.5% female) participated of whom 89% filled out both surveys, and in the second wave in September 2015, 106 students (*M*_*age*_ = 20.36, *SD*_*age*_ = 3.38, 56.6% female) participated of whom 88.7% filled out both surveys. No students participated in both waves.

### Procedure

Students, staying in rooms with measurement devices on water use, energy use and presence, received an email with a link to the online survey. As the initial response rate was rather low, a member from the research team approached students in person at the hotel and asked them to fill out the online survey.

Upon opening the online survey, participants were asked to agree to the informed consent form. Additional to a regular informed consent form, students were explicitly informed that the answers to this survey would be linked to their individual energy and water usage at The Student Hotel and were asked if they agreed with this procedure. Thereafter, participants filled in a number of questions (see Measures). After answering these questions, participants were presented with the first construal level manipulation, the “how versus why” task. Participants were then asked to indicate the ease of processing of the task and their level of self-efficacy. Students were asked to read the explanation of the initiative (see S3 Text) and finally were asked for their demographic information. After completing the first survey, participants were contacted by one of the researchers for the social distance manipulation. Participants were given the option to choose a gift (depending on the condition they were in), which they received in Week 3 of the experiment. Therefore, due to the design of the study, all participants had received both manipulations in full by the end of Week 3, which makes Week 4 the week of interest in terms of the effects of the combined construal level interventions on water and electricity use. Finally, participants were asked to fill out a post-intervention survey in week 4, which was sent to them via email.

### Manipulations

#### Construal level

Participants were asked to fill out an adjusted version of the “how versus why” task [18], which has been designed to only vary the level of abstraction at which people think about the same activity. In the low construal level condition participants were asked to list three means to reduce their water use to preserve the environment and to rate these in reference to the question “How much will engaging in this activity reduce your water use at The Student Hotel?” on a 5-point scale (1 = *a little*, 5 = *very*, *very much*). In the high construal level condition participants were asked to list three ways in which reducing their water use to preserve the environment could help reach important life goals. Participants were also asked to rate, in reference to each goal that they had listed, “How much will reducing your water use at The Student Hotel help you reach this goal?” on a 5-point scale (1 = *a little*, 5 = *very*, *very much*). Finally, participants were asked to complete a diagram, which asked them to indicate how (in the low construal level condition) or why (in the high construal level condition) they should reduce their water use (see Figures A and B in S1 Text). This way, participants in the high construal level condition were asked to think more and more abstractly about reducing their water use, whereas participants in the low construal level condition were asked to think increasingly concretely about reducing their water use [18].

#### Social distance

In order to increase participation in the experiment participants received a gift, which was non-contingent, which means that all participants received the gift irrespective of their behavior. Participants in the low social distance condition were given the option to choose a gift from four options (low social distance), and participants in the high social distance condition were given the option to choose one of four charities they wished to support (high social distance). The various gift options can be found in the S2 Text.

Participants received the gift three weeks after completing the survey, which means that in week 4 after completing the survey, all participants had received both manipulations (i.e., the construal level and the social distance manipulation). Participants in the high social distance condition received a certificate stating their name and the charitable organization they indicated to donate to at the same time as the participants in the low social distance condition received their gift. Upon receiving the gift, participants also received a message which provided a short recap of the construal level task they had received when completing the pre-intervention survey (see S3 Text).

### Measures

Unless otherwise indicated, participants scored all survey questions on 7-point Likert-type items (1 = *strongly disagree*, 7 = *strongly agree*). Information on all measured constructs in both the pre-intervention and post-intervention survey is depicted in Table 1. The constructs will be considered next.

**Table 1:** Descriptive statistics of measures from pre-intervention and post-intervention survey.

		Pre-intervention survey				Post-intervention survey			
Variable	Number of items	*n*	*M*	*SD*	α/*r*	*n*	*M*	*SD*	α/*r*
Trait construal level	10	197	5.90	2.19	-	172	6.37	2.22	-
TSH Sustainability	3	197	4.76	1.11	.766	171	4.96	1.08	.812
Environmental Self-Identity	3	197	5.09	1.01	.901	171	4.99	1.12	.953
Values									
Hedonic	3	197	5.24	1.41	.863	166	5.18	1.38	.869
Egoistic	5	197	3.84	1.43	.804	166	3.95	1.38	.828
Altruistic	4	197	5.43	1.37	.885	166	5.46	1.33	.879
Biospheric	4	197	5.15	1.51	.934	166	5.24	1.43	.926
Efficacy	3	197	5.17	0.94	.683	167	5.08	1.01	.819
Water behavior									
Shower	4	197	3.34	1.29	.711	170	3.67	1.32	.757
Shower time	2	197	10.95	5.50	.805	171	11.00	7.08	.854
Electricity behavior									
Switching off	2	197	4.36	1.67	.541	170	4.57	1.58	.598
Appliance use	4	197	5.31	1.06	.669	170	5.36	0.99	.694
Pro-environmental behavior									
Recycling	3	197	3.40	1.06	.727	170	3.45	1.00	.769
Buying envir.- friendly products	2	197	2.80	0.90	.447	170	2.83	0.98	.563
Eating meat	1	197	3.55	1.24	-	170	3.48	1.21	-

Note. All self-report measures in the pre- and post-intervention survey including sample size (n), Means (M), Standard Deviations (SD) and Cronbach’s alphas (α) or Pearson’s *r* for two-items scales.

#### Trait construal level

Participants were asked to fill out 10 items from the Behavior Identification Form (BIF; [50]), a standard scale, to measure their trait construal level. Items were selected based on relevance to students in the Netherlands, and on the correlations with the other items. Participants were asked to select one description of behavior that appeared most appropriate to them. For example, for “Painting a room” participants could choose between “Applying brush strokes” (low level, scored as 0) and “Making the room look fresh” (high level, scored as 1). The scores were summed, and higher scores thus indicated higher levels of construal.

#### Perceived sustainability

Participants rated the perceived sustainability of the student housing facility on three items (e.g., “**** is a sustainable residence”).

#### Environmental self-identity

Environmental self-identity was assessed with the scale developed by van der Werff, Steg, and Keizer [51] consisting of three items (e.g., “Acting environmentally friendly is an important part of who I am”).

#### Pro-environmental behavior

Questions were included on several pro-environmental behaviors to assess how people behaved at the student housing facility. First of all, we asked participants to rate four statements about their shower behavior (e.g., “I turn off the shower when I’m soaping myself down”). Participants were also asked to indicate their average shower time on two items (“What was the duration of your last shower?” and “How long do you shower on average?”) and their shower frequency. Secondly, we asked participants to rate four statements on their appliance use (e.g., “I wash my clothes at a lower temperature to save energy”) and two statements about switching off their appliances (e.g., “I switch appliances off instead of leaving them on standby”) as a measure of their electricity use. Finally, we asked participants to rate three questions on recycling behavior (e.g., “Do you bring glass bottles to the recycle bin?”), two questions on buying environmentally friendly products (“Are the products you buy organic?”) and one question on eating meat (“How often do you eat meat?”) on a 5-point frequency scale (1 = *never*, 5 = *always*).

#### Values

To assess personal values, participants rated items from Schwartz’s value scale [52] as “*guiding principles in their life*” on a 9-point perceived importance scale (-1 = *opposed to my principles*, 0 = *not important*, 7 = *extremely important*). We included three items for hedonic values (e.g., “Pleasure: gratification of desires”), five items for egoistic values (e.g., “Social power: control over others, dominance”), four items for altruistic values (e.g., “Equality: equal opportunity for all”) and four items for biospheric values (e.g., “Respecting the earth: harmony with other species”; [45]).

#### Ease of processing

To assess the ease of processing of the construal level task and information provided in that task, participants were asked to score whether the thought experiment was: “difficult to process/easy to process,” “difficult to understand/easy to understand,” or “difficult to comprehend/easy to comprehend” [53].

#### Self-efficacy

To assess perceived efficacy in terms of reducing energy and water use at home, participants were asked to score three items (e.g., “I feel that I know how to go about reducing my energy and water use”; [24]).

#### Demographics

Finally, we asked participants for their demographic information, including age, gender, nationality, and level of education. We also asked participants’ room numbers, with which they could access the second survey and which we used to link the measured water and electricity data to their survey answers.

#### Post-intervention survey

The post-intervention survey was mostly identical to the first survey, except that it excluded the construal level task and the demographic questions. Additionally, in the post-intervention survey we asked participants how much they liked their gift (1 = *not happy*, 5 = *very happy; M* = 4.17, *SD*_=_ 1.00) and how much they would be willing to pay for their gift in euros (*M* = 7.40, *SD* = 5.57, *n* = 126).

#### Objective measurements of energy use

Throughout the intervention period objective measurement equipment recorded individual water use, electricity use, and presence (measured with the card readers in their room). More specifically, we obtained data on a 10 -minute basis for hot water use and electricity use. Electricity use was measured at two levels: the lighting and the sockets. Finally, the card reader was used as a measure of the participant’s presence in the room. For all objective measurements we use the percentage difference in energy use (i.e., water and electricity use) as compared to the control group as our dependent variable. A detailed description of the exact use of the objective data in the subsequent analyses can be found in the S4 Text.

**Fig 1:**
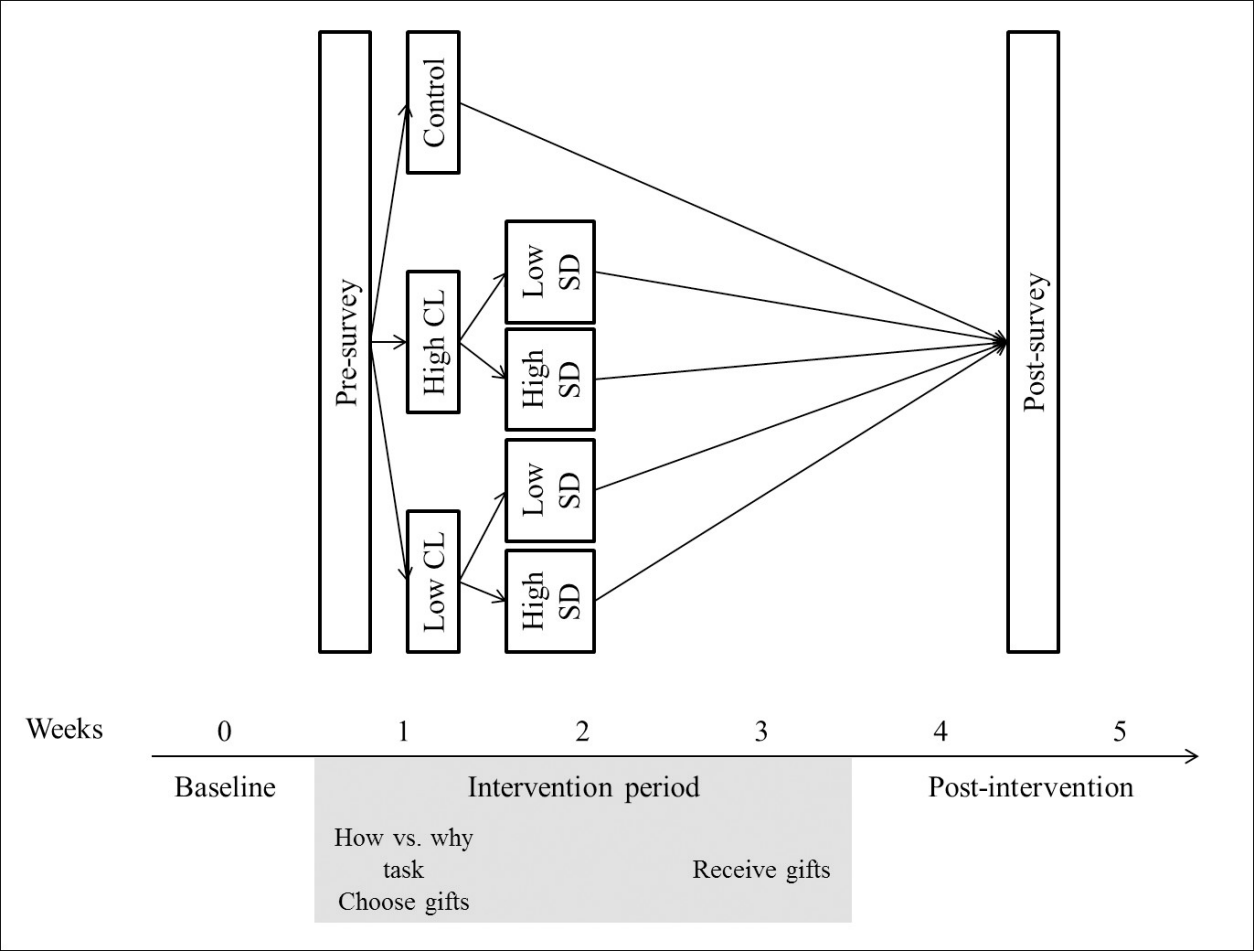
Design of experiment. CL = construal level, SD = social distance.

Fig 1 depicts the design of the study and shows which steps were taken for each week specifically. As participants received the full manipulation (by the time they had received the gift) in week 3, week 4 is the main week of interest in the following analyses.

## Results

To measure the effects of the manipulations on the dependent variables (energy or water use data), we ran a series of repeated measures analyses of variance with our within subjects variables measured at least two points in time and the manipulations of construal level and social distance as the independent between-subjects variables. In all analyses we always controlled for a number of variables: the time of data collection (wave 1 or wave 2), trait construal level (measured by the Behavior Identification Form), biospheric values, age, and gender. In the following section the effects on the objective measurements of water and electricity use data will be reported. In the SI, the correlations between the self-report variables and objective measurements of water and electricity use are reported (S4 Table), as well as the effects of the manipulations on self-report behavior (S5 Text) and supporting analyses on the objective energy use data (S1–S3 Tables).

### Target behavior

#### Six-week trend water use

**Fig 2:**
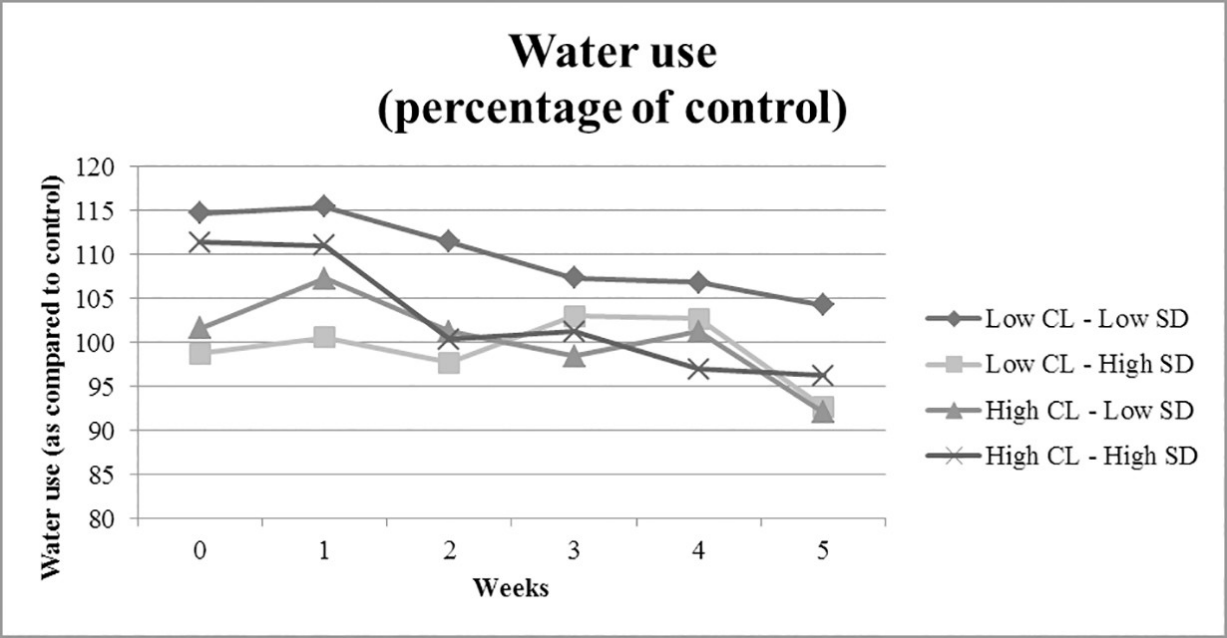
Water use depicted as percentage of control group during the six-week intervention period. CL = construal level, SD = social distance.

To test whether the experimental conditions had an (interaction) effect on the water use throughout the entire intervention period, we performed a repeated -measures analysis with six levels. Each level represents the average water use for each of the six consecutive weeks, where the first level represents the baseline week, which is the week before participants filled out the pre-intervention survey (week 0), and the subsequent weeks represent each week after filling out the survey. Participants only received the gift (i.e., to self or other) in week 3, making week 4 our main interest. However, we were also interested whether we could already observe changes before participants had actually received their gift and therefore we first analyzed the pattern of water use throughout the entire intervention period. First of all, time had a significant effect on water use throughout the six -week period, *F*(5,740) = 2.44, *p* = .033, *p*η^2^ = .016. This effect indicated that, compared to the control group, participants in the experimental conditions reduced their warm water use over the course of the experiment. Besides the main effect of time, time did not significantly interact with social distance (*F*(5,740) = 0.29, *p* = .919, *p*η^2^ = .002), construal level (*F*(5,740) = 0.47, *p* = .800, *p*η^2^ = .003), or the interaction between construal level and social distance (*F*(5,740) = 1.15, *p* = .332, *p*η^2^ = .008). Fig 2 depicts the average water use throughout the period from one week prior to the intervention to five weeks following the intervention. In addition to this analysis, we also analyzed the data per week separately and an additional graph showing the absolute water use throughout the six-week for all conditions, including the control condition (shown in S5 Table and S1 Fig, respectively).

#### Baseline vs. week 4 water use

**Fig 3:**
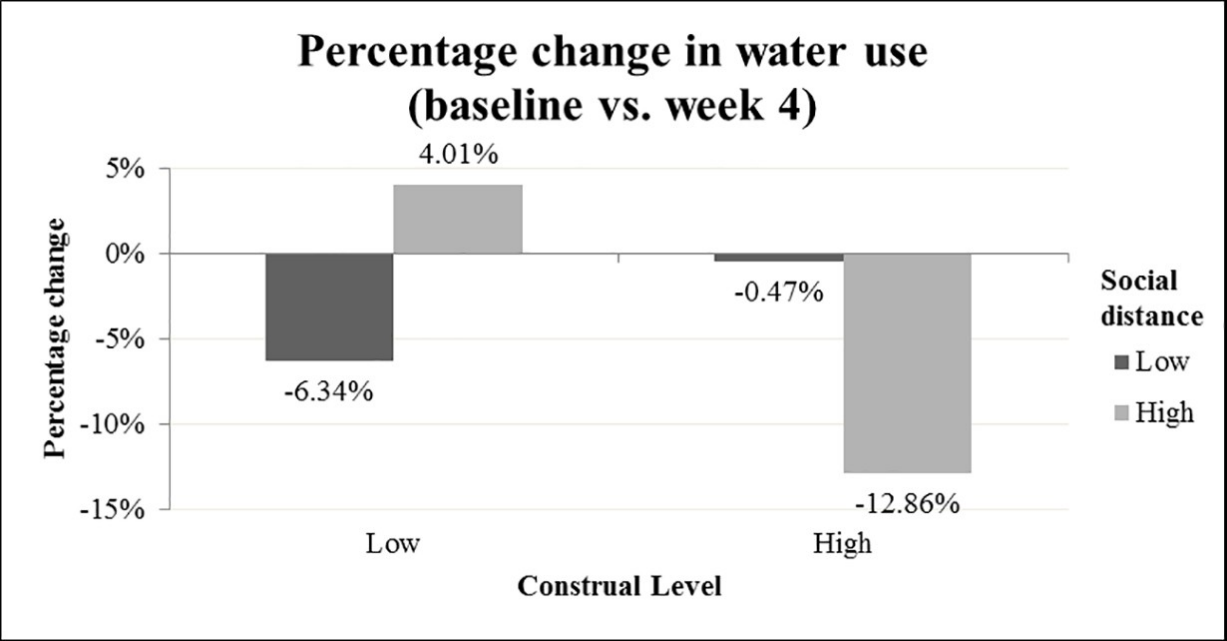
Percentage change in water use relative to control group between baseline (week 0) and week 4.

As participants received the gift in week 3 of the experiment, our main focus was on the effect of the manipulations in the fourth week after participants had filled out the first survey. Employing a repeated measures analysis with two levels (1 week prior to filling in the survey and 4 weeks after filling in the survey), we found that time did not have a significant effect on the difference in water use (*F*(1,150) = 0.60, *p* = .440, *p*η^2^ = .004), nor was there a significant interaction between time and social distance (*F*(1,150) = 0.05, *p* = .829, *p*η^2^ = .000), or time and construal level (*F*(1,150) = 0.84, *p* = .361, *p*η^2^ = .006). However, we did find a marginally significant three-way interaction effect between time, social distance and construal level (*F*(1,150) = 3.76, *p* = .054, *p*η^2^ = .024). Fig 3 depicts the interaction between construal level and social distance in percentage change compared to the control group. A difference score was computed by subtracting the water use in week 0 (the week before the intervention) from water use in week 4 (after the intervention). This difference score was used to detect whether the change in water use was significantly different between the conditions. We used contrast analysis (i.e., LSD) to detect differences between the different combinations of manipulations. First of all, in the high social distance condition (gift to other), participants who were in the high construal level condition reduced their water use significantly more than participants in the low construal level condition (*M*_*difference*_ = 18.28, *F*(1,150) = 3.91, *p* = .050, *p*η^2^ = .025). Secondly, in the low social distance condition (gift to self), we did not find a significant difference between the high and low construal level condition (*M*_*difference*_ = 6.66, *F*(1,150) = 0.57, *p* = .451, *p*η^2^ = .004). Thirdly, the social distance manipulation did not lead to significant differences in water use among participants in the high construal level condition (*M*_*difference*_ = 13.85, *F*(1,150) = 2.33, *p* = .129, *p*η^2^ = .015), nor among participants who were in the low construal level condition (*M*_*difference*_ = 11.09, *p* = .220, *p*η^2^ = .010). Besides the direct contrasts between the specific conditions, we also contrasted the congruent combinations (i.e., the high-high and low-low combinations) to the incongruent combinations (i.e., low-high combinations), and found a marginally significant difference (*M*_*difference*_ = 12.17, *SE*_*difference*_ = 6.40) between these two groups of conditions (*p* = .059). In other words, participants in the congruent construal level combinations reduced their water use more than participants in the incongruent combinations. Additionally, we found that participants in the congruent high construal level combination reduced their water use more than all other combinations (*M*_*difference*_ = 12.75, *SE*_*difference*_ = 7.42), which was marginally significant (*p* = .088). Additionally, comparing the two congruent combinations with one another, we found that there were no significant differences between the two congruent combinations (*M*_*difference*_ = 6.99, *SE*_*difference*_ = 8.63, *p* = .419). Finally, we analyzed whether the change in water use was significantly different from a 0% change. As such, only participants in the congruent high construal level combination condition (i.e., high social distance and high construal level) showed a significant reduction in their water use as compared to no change (*t*(150) = 2.25, *p* = .026, *p*η^2^ = .033). The other combinations did not significantly differ from a 0% change (low social distance and low construal level (*t*(150) = -1.20, *p* = .233, *p*η^2^ = .009); low social distance and high construal level (*t*(150) = -0.10, *p* = .918, *p*η^2^ = .000); high social distance and low construal level (*t*(150) = 0.58, *p* = .565, *p*η^2^ = .002)). These results provide only partial support for Hypothesis 1 and show that the congruent high construal level combination is effective, especially compared to incongruent combinations, but is not significantly more effective than the congruent low construal level combination.

### Biospheric values as a moderator

Biospheric values have been pinpointed as a predictor of pro-environmental behavior. To test our two competing hypotheses, we explored the potential moderating effect of biospheric values and added biospheric values to the repeated measures analysis.

#### Baseline vs. week 4 water use

The repeated measures analysis with two levels (1 week prior to filling in the survey and 4 weeks after filling in the survey) indicated that time did not have a significant effect on water use (*F*(1,147) = 1.00, *p* = .319, *p*η^2^ = .007) nor did time interact with social distance (*F*(1,147) = 2.14, *p* = .145, *p*η^2^ = .014), construal level (*F*(1,147) = 0.75, *p* = .387, *p*η^2^ = .005), social distance and biospheric values (*F*(1,147) = 2.16, *p* = .144, *p*η^2^ = .014), or construal level and biospheric values (*F*(1,147) = 0.48, *p* = .492, *p*η^2^ = .003).

**Fig 4:**
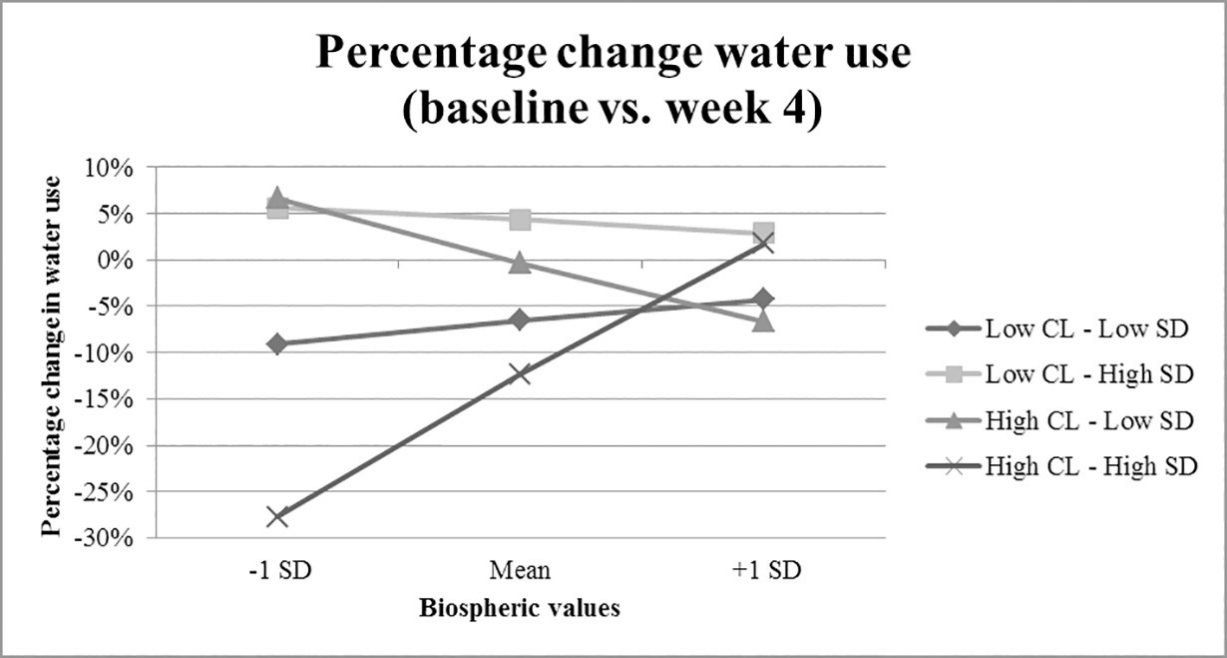
Percentage change in water use between baseline (week 0) and week 4 with biospheric values as moderator. SD = social distance, CL = construal level.

The analysis did reveal a significant three-way interaction between time, social distance and construal level (*F*(1,147) = 6.10, *p* = .015, *p*η^2^ = .040) and a four-way interaction between time, social distance, construal level and biospheric values (*F*(1,147) = 4.37, *p* = .038, *p*η^2^ = .029). This interaction showed that biospheric values affected the effectiveness of the construal level manipulation in combination with the social distance manipulation. Using the PROCESS macro by Hayes [54], we further analyzed the three-way interaction between the experimental conditions and biospheric values on water use. People who scored high on biospheric values (mean +1 SD) were not affected by the combination of manipulations, as the interaction between time, social distance and construal level was not significant (*F*(1,147) = 0.00, *p* = .970, *p*η^2^ = .000). In contrast, participants who scored lower on biospheric values (mean -1 SD) were influenced by the combination of manipulations, as the interaction between time, social distance and construal level remained significant (*F*(1,147) = 7.85, *p* = .006, *p*η^2^ = .051). For people who scored lower on biospheric values, we found the following effects of the interaction between the construal level manipulation and the social distance manipulation. First of all, the construal level manipulation had a significant effect on water use among participants in the high social distance condition (*t*(147) = -2.82, *p* = .005). More specifically, participants who were in the high construal level condition reduced their water use significantly more than participants in the low construal level condition (see Fig 4.). Secondly, the construal level manipulation did not have a significant effect among participants in the low social distance condition (*t*(147) = 1.26, *p* = .211). Thirdly, social distance had a significant effect on reduction in water use among participants who were in the high construal level condition (*t*(147) = 2.85, *p* = .005). Particularly, participants in the high social distance condition reduced their water use significantly more than participants in the low social distance condition. Finally, social distance did not have a significant effect on the reduction in water use among participants who were in the low construal level condition (*t*(147) = -1.22, *p* = .225). The interaction between biospheric values and the experimental conditions is depicted in Fig 4, showing the percentage change from baseline to week 4.

Besides the moderating effect of biospheric values, construal level theory poses that people are more inclined to act upon their values when they think at a high construal level. Next to the moderation analysis on change in water use, we were interested in the correlation between biospheric values (measured on the pre-intervention survey) and absolute water use. We therefore specifically tested whether the correlation between biospheric values (measured on the pre-intervention survey) and water use was stronger among participants in the congruent high construal level combination. Across all participants, baseline water use did not correlate with biospheric values (Pearson’s *r* = -.011, *p* = .884), nor did biospheric values correlate with water use in week 4 (Pearson’s *r* = .093, *p* = .205). However, when we looked at the specific correlations between water use and biospheric values for the high congruent construal level combination, we found that although biospheric values did not significantly correlate with baseline water use (Pearson’s *r* = .044, *p* = .783), biospheric values did significantly correlate with water use in week 4 (Pearson’s *r* = .421, *p* = .008). Across all other combinations, biospheric values did not significantly correlate with water use either at baseline or in week 4 (*p* > .05). This correlation thus indicates that people act more upon their values when they think at a high construal level.

### Spillover behavior

#### Baseline vs. week 4 electricity use

To test the effect of the experimental conditions on electricity use, we also looked at the difference in electricity use between the week before filling out the survey and week 4 after filling out the survey.

*Sockets*. The repeated measures analysis of variance showed that time did not have a significant effect on socket use (*F*(1,130) = 1.22, *p* = .272, *p*η^2^ = .009). Moreover, we did not find a significant interaction with time on socket use for construal level (*F*(1,130) = 0.01, *p* = .931, *p*η^2^ = .000), or for the interaction with construal level and social distance (*F*(1,130) = 0.20, *p* = .655, *p*η^2^ = .002). Social distance did significantly interact with time (*F*(1,130) = 5.84, *p* = .017, *p*η^2^ = .043), which showed that participants in the low social distance condition increased their socket use (*M*_*pre*_ = 117.09, *SE*_*pre*_ = 7.66; *M*_*post*_ = 118.75, *SE*_*post*_ = 7.39), whereas participants in the high social distance condition reduced their socket use (*M*_*pre*_ = 118.93, *SE*_*pre*_ = 9.08; *M*_*post*_ = 95.38, *SE*_*post*_ = 8.76). Similar to water use, we also compared the congruent high construal level combination with the other combinations. We found a marginally significant difference, showing that those in the congruent high construal level combination condition reduced their socket use more than the other combinations (*M*_*difference*_ = 22.57, *SE*_*difference*_ = 12.43, *p* = .072).

*Light*. The analysis revealed no significant interactions between time and construal level (*F*(1,130) = 2.15, *p* = .145, *p*η^2^ = .016), nor between time, social distance and construal level (*F*(1,130) = 0.26, *p* = .612, *p*η^2^ = .002). In contrast to socket use, time did have a significant effect on light use (*F*(1,130) = 7.19, *p* = .008, *p*η^2^ = .052), which showed that participants across all experimental conditions decreased light use from baseline (week 0; *M* = 126.78, *SE* = 6.34) to week 4 after the intervention (*M* = 115.25, *SE* = 5.57). We also found a significant interaction between time and social distance (*F*(1,130) = 3.90, *p* = .050, *p*η^2^ = .029), showing that participants in the high social distance condition decreased their light use (*M*_*pre*_ = 131.25, *SE*_*pre*_ = 9.85; *M*_*post*_ = 110.79, *SE*_*post*_ = 8.61) significantly more than participants in the low social distance condition (*M*_*pre*_ = 122.31, *SE*_*pre*_ = 8.31; *M*_*post*_ = 119.70, *SE*_*post*_ = 7.26). In contrast to water and socket use, when we contrasted the congruent high construal level combination to the other combinations, we found no significant difference in terms of change in light use (*M*_*difference*_ = 7.39, *SE*_*difference*_ = 10.89, *p* = .499).

## Discussion

This study was designed to investigate the effects of a combination of construal level manipulations on warm water use. Moreover, we looked at the possible moderating effect of biospheric values on water use. Additionally, we were interested in the net environmental impact, which is why we also tested the effect of the two construal level manipulations on spillover to electricity use.

### Congruent construal level combinations

#### Target behavior

Our main variable of interest in this study was the warm water use of participants. First off, we expected that participants who received a combination of manipulations that were at the same level of construal–either both at a high level of construal or both at a low level of construal–would be more effective than combinations of manipulations that were not aligned in terms of construal level (Hypothesis 1). Our results on the measured warm water use after the intervention (i.e., week 4) are partially in line with this expectation. Specifically, we found that the congruent combinations led to a marginally significant larger reduction in water use as compared to the incongruent combinations. Participants who received a combination of a high and low construal level manipulation did not significantly change their water use when we compared their change in water use to 0% change. Moreover, we found that a combination of two high-level construals was most effective in terms of motivating people to reduce their warm water use as compared to no change. Less effective, however, was the combination of two low-level construals, which did not differ from a 0% change. These results add to previous findings on construal level manipulations by showing that especially aligned high construal levels are effective when targeting warm water use. Due to the nature of the experiment, we could not specifically test how efficiently participants processed the combination of interventions. We speculate that the congruent combinations are processed more fluently [26]. Moreover, when the two manipulations were at the same construal level, we believe that the manipulations enforce one another, whereas behavior in the incongruent combinations are solely driven by the low construal level component.

The high construal level combination was most effective in our experiment, while the low construal level combination did not lead to a significant reduction in warm water use. One explanation for the ineffectiveness of the low construal level combination could be the fact that we targeted curtailment behavior, which we measured during multiple weeks. It could be argued that both aligned combinations are processed more efficiently, but that the low construal level combination was not effective in this longer-term situation. It could be that the low construal level combination is effective when one-off decisions are being targeted, as has been found in previous studies [24,27,28]. The fact that the combination of two high construal levels was most effective in this experiment may have been due to the type of behavior that was being targeted. While it has been suggested that high construal levels are more effective than low construal levels when targeting pro-environmental behavior [10], this premise has not been extensively tested in either the lab or the field in direct reference to pro-environmental behavior. Our study provides initial evidence for the fact that high construal level approaches can indeed be more effective when targeting pro-environmental curtailment behavior. In sum, our findings suggest that a high construal level approach is only effective when combined with another high construal level approach and not when combined with a low construal level approach. In the latter case, the low construal level component may become the driving factor for behavior, which may not be very effective when targeting pro-environmental behavior.

In terms of the ineffectiveness of incongruent approaches, we believe that the low construal level component makes people consider the more immediate consequences of their behavior and evaluate the high construal level component in this light. As such, in the incongruent combination, participants in the low construal level condition (i.e., those exposed to the “how” task) were given the opportunity to choose to donate to a charitable organization. It could be that participants justified their lack of behavior change on the basis of their “good” deed of donating and, indeed, participants in this combination valued their gift the most. In the other incongruent combination, participants were asked to think about their water use at a high construal level and were subsequently given a gift to self. The latter manipulation made participants solely focus on their concrete, day-to-day considerations, rather than the overall impact of their behavior at a higher construal level. The way participants were asked to think about their water use was not in line with the type of gift they received and therefore was not effective in changing their behavior.

#### Biospheric values

We expected that biospheric values would moderate the effects of our manipulations in one of two ways. On the one hand, we anticipated that people with high biospheric values would be more affected by our high construal level manipulations, as they would act more upon their inner values. On the other hand, we also theorized that people with high biospheric values could be less affected by our manipulations in general, as there is less room for improvement in their current behavior. Our results indicate that participants who scored lower on biospheric values were actually mostly influenced by the aligned high construal level manipulations. This is in contrast with construal level theory, which suggests that people act more upon their inner values when they think at a high rather than low construal level. Important to note, however, is that participants who scored lower on biospheric values still scored above the midpoint of the scale, indicating that they do care for the environment to some extent. Participants who scored highest on biospheric values hardly changed their water use, which could be due to a “ceiling effect.” As such, previous studies [51] show that people who score higher on biospheric values also portray more pro-environmental behavior and, therefore, they may have reached a limit in how much they can still change. The fact that we did not find a significant correlation between biospheric values and objectively measured water use, but did find a significant correlation between biospheric values and self-reported energy use behavior, is interesting and important in itself. As most previous studies have found a significant correlation between biospheric values and self-reports, a lack of correlation with objective data is potentially problematic for predicting actual behavior on the basis of biospheric values. It therefore seems, as Klöckner [55] argues as well, that other moderating and mediating factors influence the link between biospheric values and energy use behavior, especially when the latter is measured objectively. Note however, that we did find that participants in the congruent high construal level combination acted more upon their values in terms of their water use in week 4 (as shown by the significant correlation). This finding is in line with construal level theory, which poses that people act more upon their values when thinking at a high construal level. We consider the relationship between biospheric values and both objectively and self-reported energy use an important one that would be interesting to study in the future, for example, by running a meta-analysis.

Another explanation for the effectiveness of the high construal level combination, could be the goal that was highlighted in the direct construal level manipulation. As such, the direct construal level manipulation included an environmental goal in both the high and low construal level condition. When people think of this at a high construal level, they may act more upon these values, whereas at a low construal level values are not the driving factor of their behavior. As suggested, values can be made temporarily salient [44], which may have been the case when two high construal level approaches were combined. In other words, environmental values may have been made salient and people may thus have acted upon these emphasized values. As noted, participants who scored lower on biospheric values still scored above the midpoint of the scale, indicating that they do care for the environment to some extent. Whether the same results hold for participants who indicate not to value the environment at all remains to be studied. Future research should look more into the role of values in direct reference to the effectiveness of interventions and other programs targeted at motivating people to act in a pro-environmental manner, especially among those who do not strongly endorse biospheric values or already act in a pro-environmental manner.

#### Spillover

In terms of spillover behavior, we expected that only the combination of two high-level construals would lead to positive spillover behavior (Hypothesis 2). This expectation is partially supported by the spillover to socket use, as our results indicated that the congruent high construal level combination marginally significantly differed from the other combinations. The results for light use did not support Hypothesis 2, as the high construal level combination did not significantly differ from the other combinations. Our results, however, do indicate that the social distance manipulation by itself, irrespective of the construal level manipulation, had a significant effect on spillover to electricity use. More specifically, participants in the high social distance condition reduced their electricity use in terms of socket and light use more than participants in the low social distance condition. This is in line with earlier work by Evans et al. [42] showing that appealing to self-transcendent values can lead to positive spillover. They argue that spillover occurs because important values are appealed to and this ultimately drives behavior. In terms of construal level theory, this argument would provide a similar explanation; appealing to self-transcendent values may lead to more abstract thinking and makes people see the similarities between different types of behaviors. Moreover, as people usually want to be consistent in their behavior [56], they may, therefore, also be motivated to change their behavior in other areas. The fact that the direct construal level manipulation did not affect electricity use should be investigated in future work, in order to gain understanding in how different types of construal level manipulations affect spillover behavior.

### Implications

This research provides an explanation for which combinations of interventions may work especially well when targeting pro-environmental behavior. As previous studies have shown mixed results on a multitude of combinations of intervention approaches, we wanted to take a closer look at the potential underlying factors that may determine the effectiveness of combined manipulation approaches. Especially for the target behavior, construal level theory provides a possible explanation for when some combinations do and do not work well together. Practically this explanation suggests that when, for example, designing intervention programs the construal level of the different types of manipulations should be considered. More specifically, our findings suggest that congruent high construal level combinations are most effective when targeting pro-environmental curtailment behavior, and more specifically (warm) water use. In contrast, combinations that combine a high and low construal level were not effective, which suggests that these combinations should be avoided when designing campaigns targeting pro-environmental behavior. Moreover, spillover was also most likely to occur among participants in the high social distance condition, which is associated with a high construal level. Findings from our study, therefore, suggest that high construal level interventions should be favored over lower construal level interventions, as the high construal level manipulations have a greater effect on the target behavior as well as on other pro-environmental behavior. Moreover, the effects of these high construal level interventions were especially present for participants with lower biospheric values, which means that this is also a valid approach to motivate people who may not already act in a pro-environmental manner.

### Limitations and future research

The unique living lab design of our study allowed for many analyses that are otherwise impossible to do. In particular, we could specifically target individual behavior, look at individual differences in direct reference to actual individual energy use and control for whether people were present or absent. This way, we could also see that objective and self-report measures are correlated to some extent (see S4 Table), but that there is a gap. This is in line with previous research [57], which can be due to different reasons. For example, people may not be very capable of indicating how much water and energy they use at home, as it is a vague and abstract concept to them [58,59]. As large proportions of studies in this field rely solely on self-reports, future research should study whether interventions result in similar patterns when using objective behavioral measures.

Despite the advantages of our design, there are some downsides to the design of this study as well. First of all, we ran our study with a student population, who mostly live by themselves for the first time in their lives. This may have affected how susceptible they are to interventions, as it has been found that people are more likely to change their habits when they have just moved [60]. This raises the question of whether the effects of our intervention can be translated to more stable household settings as well. Nonetheless, this group may be particularly interesting as they constitute the energy users of the future. Another difference with standard household settings is that people usually pay for their energy bill, which was not the case in our experiment as the room rent includes energy and thus eliminates the possibility that monetary concerns guide the behavior change. While the elimination of financial motivations is a specific feature of our “living lab,” this is different from settings where people do have financial incentives to change their behavior and should be taken into account when generalizing these findings. That said, previous work has shown [6] that people care about monetary benefits, irrespective of whether these benefits are made salient or not. This could actually suggest that our manipulations might even be more effective in situations where people do have monetary benefits of acting in a pro-environmental manner, as people can also rationalize their behavior on monetary accounts later on [61]. Moreover, a large portion of individual’s energy use also occurs in situations where they do not directly pay for their individual energy use, such as at work and in public buildings [62]. Future research should explore how a similar intervention would work in different settings, such as in an office building or in a household setting.

Another factor in this research that may have influenced behavior is the fact that the people participating in this study were aware of being monitored. Obviously, this may influence the way they acted. However, the fact that participants in the control condition received the exact same information (in terms of the surveys), but not the manipulations, allowed us to control for potential effects that are due to the notion of being monitored. Although we used a control group to control for the potential monitoring effects, it would be of interest to see whether similar results would arise when people are unaware of the fact that they are being monitored. Moreover, in our experiment participants did not explicitly receive information on how much energy they used throughout the experiment, which may make the monitoring issue less salient throughout, as compared to studies using feedback for example [63]. Additionally, compared to other intervention studies, our study was less prone to self-selection of participants, as participants were simply approached based on the fact that they were randomly placed in the rooms with measurement equipment and not based on their willingness to participate. Besides, this study was not communicated to participants as a clear energy saving program and it is therefore unlikely that people decided not to participate based on the purpose of the research.

In this study we tested the effect of combinations of construal manipulations on curtailment behavior (i.e., warm water use) and find that especially high construal level combinations are effective. In order to generalize these findings to other types of behaviors that are beneficial to the environment, such as one-off investment decisions, future research is needed. Previous research has shown that low construal level approaches can be effective when targeting one-off decisions (e.g., tax breaks on electric car purchases), but the question often remains whether this has a lasting positive effect on behavior. Future research should investigate whether high construal level combinations are also more effective when trying to stimulate one-off (investment) decisions, or whether a different approach is more effective.

Finally, we tested two manipulations of construal level, one of which is rather established as a construal level manipulation (viz., the “how versus why” task), whereas the social distance manipulation (viz., the gift to self or other) is less established as such. We argued that the gifts affected the experienced social distance and indirectly people’s construal level. However, other explanations can be posed for the congruency between the construal level manipulation and our social distance manipulation. As such, as noted in the introduction, the gifts may have evoked different types of values (i.e., self-interest or pro-social values). We acknowledge that such values could have been at play at the same time but believe that the directionality of the effects would be the same from a values perspective. Moreover, similar to our social distance reasoning and in line with previous work [28], we believe that pro-social values are associated with high construal level thinking and self-interest values are more in line with low construal level thinking. We acknowledge that we are unable to tease the effect of self-interest and pro-social values apart from the associated construal level. Even though we suggest that this will not have affected the directionality of the effects, pure construal level manipulations may have led to different effects. In order to tease these two constructs apart, future work could for example focus solely on pro-social values and either represent them at a high construal level or a low construal level. Moreover, it should be tested in future work whether other operationalizations of construal level manipulations have a similar effect on energy saving behavior. Theory suggests that the same effect would emerge when other (combinations of) construal manipulations are used. However, there is simply not enough empirical evidence to make this claim at this point [20]. Therefore, future research should explore the opportunities of combining, for example, a temporal construal manipulation with a hypothetical construal level manipulation. Moreover, it would be very valuable to qualify previous studies based on their construal level and assess whether indeed aligned combinations are most effective in case long-term curtailment behavior is targeted.

## Concluding remarks

The aim of this research was to investigate the impact of combinations of intervention approaches. Our research setting allowed us to test the effect of a combination of construal level manipulations on an individual level with objective measurements of energy use. Our findings show that construal level theory can provide a possible explanation for which combinations are most effective. As pro-environmental behavior is about more than just one behavior or just a one-off decision, we studied the effect of our intervention on both the target behavior and other pro-environmental behaviors. Our findings suggest that a congruent combination at a high construal level has the largest effect on the target behavior and that spillover is most likely to occur when a high construal level approach is used (viz., high social distance). Future work should study how different combinations, based on their level of construal, affect pro-environmental behavior. In sum, our study suggests that when designing interventions one should consider the level of construal of the individual components of the intervention in order to find the most effective way to target pro-environmental behavior.

## Supporting information

S1 Text Construal level manipulations. (PDF)

S2 Text Social distance manipulations. (PDF)

S3 Text Initiative of experiment explained. (PDF)

S4 Text Use of objective energy data for analyses. (PDF)

S5 Text Results repeated measures analyses on self-report measures. (PDF)

S6 Text Survey questions–Not previously published. (PDF)

S1 Table The interaction between the covariates and time in the repeated measures analyses (p-values are reported). (PDF)

S2 Table Main effects in repeated measures analyses on average energy and water use. (PDF)

S3 Table Main effects in repeated measures analyses on the average self-report measures. (PDF)

S4 Table Correlations between self-reported values and measures warm water and electricity use during baseline. (PDF)

S5 Table The effect of time and the interaction between time and the independent variables in the repeated measures analysis on objectively measured water use. (PDF)

S1 Fig Absolute change in water use throughout 6-week intervention period compared to baseline. (PDF)
