# Supplementary material for: Which construal level combinations generate the most effective interventions? A field experiment on energy conservation
Source: PLoS One. 2019 Jan 17;14(1):e0209469. doi: 10.1371/journal.pone.0209469 (PMC6336225; doi:10.1371/journal.pone.0209469)

S1 Text. Construal Level Manipulations.

Low construal level condition. Participants in the low construal level condition were asked to consider *how* they could reduce their water use. Participants were asked to read the following instructions:

For everything we do, there always is a process of how we do it. Moreover, we can often follow our broad life-goals down to our very specific behaviors. For example, like most people you probably hope to find happiness in life. How can you do this? Perhaps finding a good job, or being educated, can help. How can you do these things? Perhaps by earning a college degree. How do you earn a college degree? By satisfying course requirements. How do you satisfy course requirements? In some cases, you have to study to pass the course's exam. Research suggests that engaging in thought exercises like that above, in which one thinks about how one's ultimate life goals can be expressed through specific actions, can improve people's life satisfaction. In this study, we are testing such a technique. This thought exercise is intended to focus your attention on how you do the things you do. For this thought exercise, please consider the following activity: "reducing your water use at The Student Hotel to preserve the environment."

High construal level condition. In the high construal level condition participants read a similar passage as the one for the low construal level condition, but with a focus on *why* they would reduce their water use at The Student Hotel to reach important life goals. They read the following instruction:

For everything we do, there always seems to be a reason for why we do it. Moreover, we can often trace the causes of our behavior back to broad life-goals that we have. For example, you might currently be working on an assignment for a course you're following. Why are you doing this? Perhaps to satisfy a course requirement. Why are you satisfying the course requirement? Perhaps to pass the course. Why pass the course? Perhaps because you

want to earn a college degree. Why earn a college degree? Maybe because you want to find a good job, or because you want to educate yourself. And perhaps you wish to educate yourself or find a good job because you feel that doing so can bring you happiness in life. Research suggests that engaging in thought exercises like that above, in which one thinks about how one's actions relate to one's ultimate life goals, can improve people's life satisfaction. In this study, we are testing such a technique. This thought exercise is intended to focus your attention on why you do the things you do. For this thought exercise, please consider the following activity: "reducing your water use at The Student Hotel to preserve the environment."

Diagrams. Below are the diagrams participants were asked to complete.

Fig A. Diagrams for high (left) and low (right) construal level manipulations.

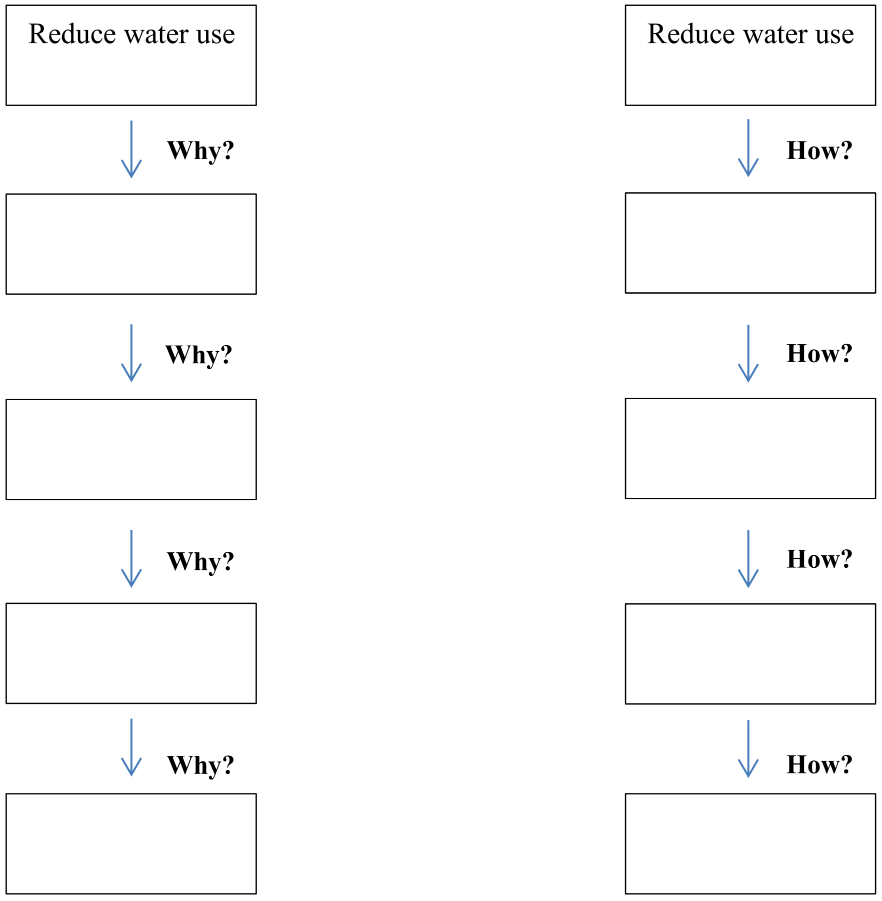

Supplement: S1 Text — (PDF) [file pone.0209469.s001.pdf]
