# Supplementary material for: Which construal level combinations generate the most effective interventions? A field experiment on energy conservation
Source: PLoS One. 2019 Jan 17;14(1):e0209469. doi: 10.1371/journal.pone.0209469 (PMC6336225; doi:10.1371/journal.pone.0209469)
Supplement: S2 Text — (PDF) [file pone.0209469.s002.pdf]

**S2 Text. Social distance manipulations.**

**Fig A. Gift options in low social distance condition.**

---

The management of The Student Hotel would like to thank you for participating in our new water conservation initiative.

To show you how much we value and appreciate your efforts in helping us to reduce our environmental impacts, we have organized a gift for you. You can choose one gift from the four items below.

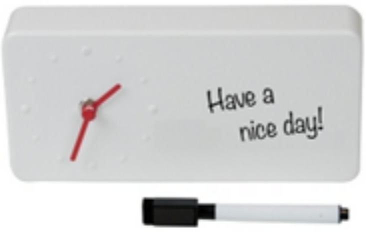


**Memo alarm clock**
Pimp your alarm clock with this Memo alarm clock. You can write or draw on this white alarm clock! This way you can leave memos or use your creativity and draw your own design.

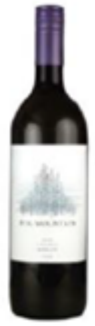

**A bottle of wine**
You can choose for a bottle of red or a bottle of white wine

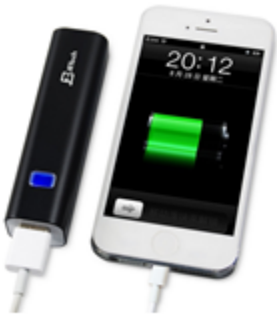


**Portable External Battery Charger**
Recharge your phone or other devices with this small external battery charger by enabling charge speeds of up to 1 amps, add more than a full charge or 4 hours of additional talk time.

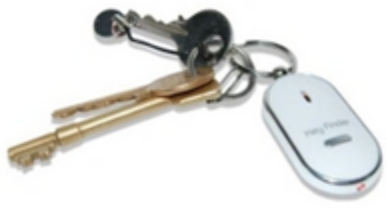

**Key finder**
Never lose your keys again with this key finder. You just whistle and this gadget will light up and make sound.

---

**Fig B. Gift options in high social distance condition.**

The management of The Student Hotel would like to thank you for participating in our new water conservation initiative.

To show how much we value and appreciate your efforts in helpin us to reduce our environmental impacts, we have organized to give a donation on your behalf to a not-for-profit organization. You can choose from one of the causes below you wish to support.

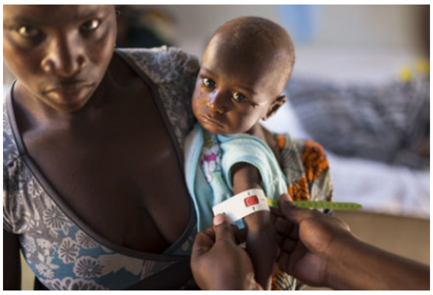

**Provide a malnourished child with extra nutritious peanut paste for two weeks**
Every day children die because they are malnourished. With this peanut paste they gain weight quickly and increase their resistance to diseases

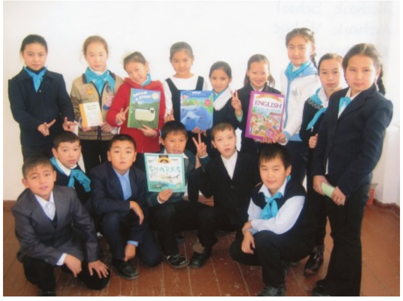

**Fund the shipping costs of approximately 5 books from the US to organizations who request them**
These include requests from Peace corps volunteers, libraries and schools all over the world

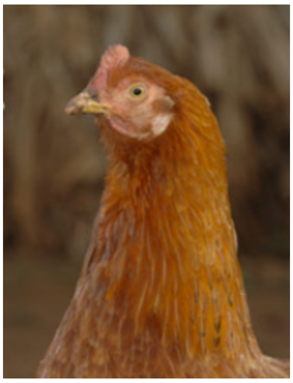

**One chicken for families in Ethiopia, Lesotho, Uganda or Kenya**
Chickens provide meat and up to 200 eggs a year - a vital source of protein and income

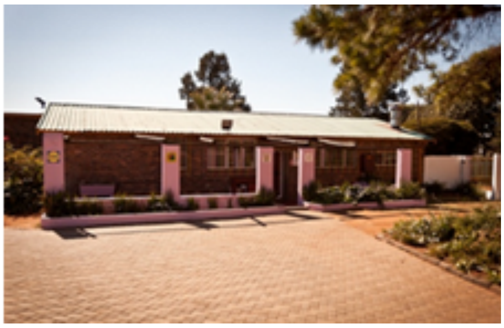

**Provide education for one child for 6 months**
With this donation a child in South Africa can go to school for 6 months
