# Supplementary material for: Which construal level combinations generate the most effective interventions? A field experiment on energy conservation
Source: PLoS One. 2019 Jan 17;14(1):e0209469. doi: 10.1371/journal.pone.0209469 (PMC6336225; doi:10.1371/journal.pone.0209469)

S3 Text. Initiative of experiment explained.

The Student Hotel as a sustainable residence

The Student Hotel is committed to being a sustainable residence. Here at The Student Hotel there are a number of efforts in place to ensure that their carbon footprint is as low as possible. These include investing in energy efficient technology and infrastructure and providing eco-friendly transport for residents.

We ask that you help us to further reduce our environmental impact by participating in our new water conservation initiative. In minimizing your water use in the shower/bath as much as you possibly can, you will help us to ensure that our environmental impact is minimal.

Recap
In this survey we asked you to complete a diagram on why it is important to reduce your water use. Of course, there were no right or wrong answers in this exercise. Below you can find two examples of filled in diagrams from previous research. These diagrams show possible reasons for why it can be important to reduce your water use. [high level]

In the survey we asked you to complete a diagram similar to the diagram below. Below you can find two examples of filled in diagrams. Of course, there were no right or wrong answers in this exercise. The two examples below come from previous research and show possible ways in which you could reduce your water use. [low level]

Fig A. Example diagrams of low construal level condition.

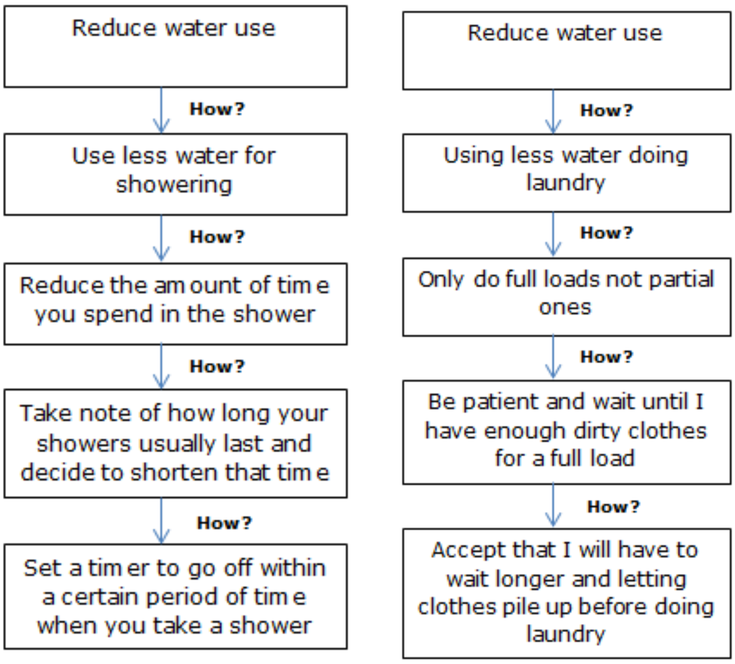

Fig B. Example diagrams of high construal level condition.

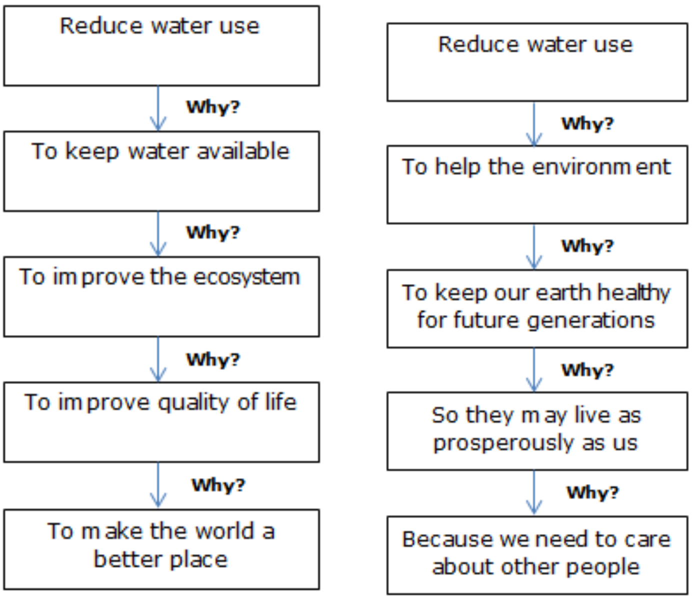

Supplement: S3 Text — (PDF) [file pone.0209469.s003.pdf]
