# Supplementary material for: Which construal level combinations generate the most effective interventions? A field experiment on energy conservation
Source: PLoS One. 2019 Jan 17;14(1):e0209469. doi: 10.1371/journal.pone.0209469 (PMC6336225; doi:10.1371/journal.pone.0209469)
Supplement: S4 Text — (PDF) [file pone.0209469.s004.pdf]

S4 Text. Use of objective energy data for analyses.

In terms of water use, we obtained data on individual hot water usage on a 10 minute basis. Electricity use was measured at two levels; one measurement device measured the automatic lighting in the room which could only be activated when the student's card was in the reader and another device measured the use of the power outlets in the room, which could be used at all times. The electricity data was also measured at intervals of 10 minutes. Finally, the card reader allowed us to see when people were actually in the room. With this measure we could account for individual differences in room use and thus their actual behavior when they were at home.

For analyzing the water data we have computed average water use per week. As participants were not present on all days during the week, we computed the average daily water use for each specific week. We included the water use for a specific day when participants had used more than 5 liters of warm water, as an indication of being present. Outliers (> 2 *SDs*) were replaced with 2 standard deviations from the mean and missing values (due to software problems) were replaced by means of linear interpolation (viz., the average of the two adjacent weeks). Subsequently, in line with Asensio and Delmas (60), we compared water use with the control group who did not receive any treatment to rule out other influences beyond the intervention. As such, the average daily water use per week per participant is calculated and divided by the average daily water use for that week for the control group, multiplied by a hundred. This number indicates the relative warm water use compared to the control group. This approach ensures that regression to the mean dynamics are controlled for, as we specifically compare the water use to a group that did not receive the treatment, but was aware of being monitored.

For analyzing the electricity data, we used the presence data detected by the card reader as the indicator for how long people were in the room. We calculated average electricity use per

hour present for each week of interest. Similar to the water use, outliers were replaced with two standard deviations from the mean and missing values were replaced by means of linear interpolation. Again, the electricity use was also compared to the control group. As such, the average hourly electricity use per week is divided by the average hourly electricity use for the control group, and multiplied by a hundred.
