# Supplementary material for: Which construal level combinations generate the most effective interventions? A field experiment on energy conservation
Source: PLoS One. 2019 Jan 17;14(1):e0209469. doi: 10.1371/journal.pone.0209469 (PMC6336225; doi:10.1371/journal.pone.0209469)
Supplement: S5 Text — (PDF) [file pone.0209469.s005.pdf]

S5 Text. Results repeated measures analyses on self-report measures.

Next to the objective measures of water and electricity use, participants were asked to score several statements on their behavior at The Student Hotel. Besides questions on water use behavior, we also asked participants to rate to what extent they engaged in other energy-related behaviors at home, including appliance use and switching off behavior. We ran a repeated measures analysis with the pre- and post-scores as the two levels in the analysis. Additionally, similar to the analyses on the objective measures of energy use, we controlled for wave, age, gender, biospheric values and trait construal level (i.e., BIF).

Self-reported shower behavior. A repeated measures analysis with two levels (pre-intervention and post-intervention score on shower behavior) revealed that time did not have a significant effect on self-reported shower behavior ($F(1,141) = 0.00$, $p = .977$, $p\eta^2 = .000$). There was a significant interaction between time and social distance on self-reported shower behavior ($F(1,141) = 6.15$, $p = .014$, $p\eta^2 = .042$); participants in the low social distance condition improved on their shower behavior ($M_{pre} = 3.21$, $SE_{pre} = 0.14$; $M_{post} = 3.83$, $SE_{post} = 0.14$), whereas participants in the high social distance condition only slightly improved on their shower behavior ($M_{pre} = 3.35$, $SE_{pre} = 0.15$; $M_{post} = 3.54$, $SE_{post} = 0.15$). There was not a significant interaction between time and construal level ($F(1,141) = 0.28$, $p = .595$, $p\eta^2 = .002$) or between time, construal level and social distance ($F(1,141) = 1.84$, $p = .177$, $p\eta^2 = .013$).

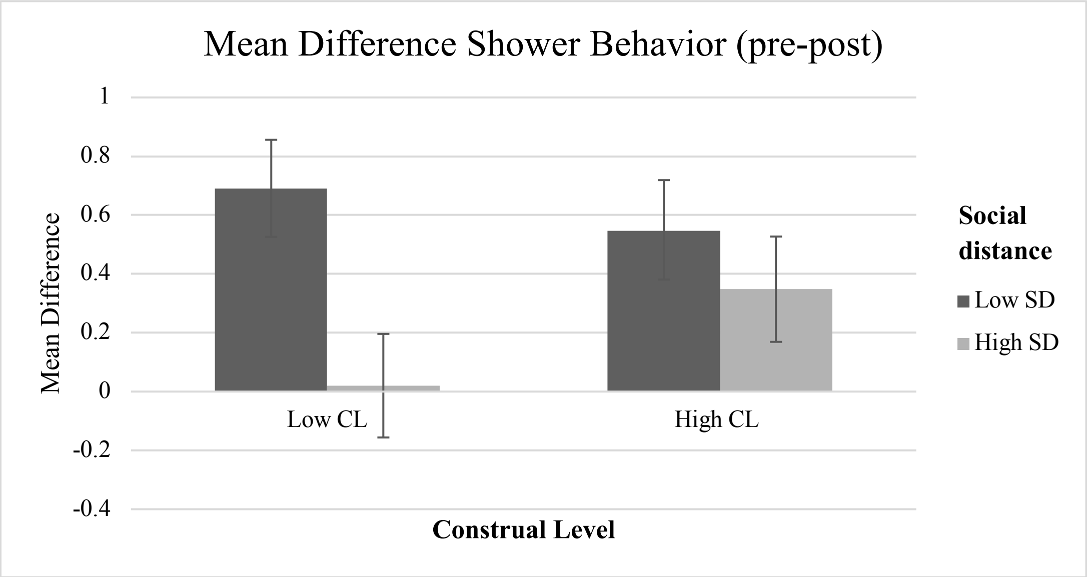

Fig S5.1. Mean difference shower behavior (post shower behavior minus pre shower behavior). Higher scores indicate that participants improved on shower behavior. Error bars represent ± 1 SE.

Self-reported shower time. Participants were also asked for their average shower time and we performed a repeated measures analysis with two levels (pre-intervention and post-intervention shower time). The analysis revealed that construal level significantly interacted with time ($F(1,142) = 6.09$, $p = .015$, $p\eta^2 = .041$), which indicated that participants in the high construal level condition reduced their self-reported shower time ($M_{pre} = 10.75$, $SE_{pre} = 0.65$; $M_{post} = 9.87$, $SE_{post} = 0.82$), whereas participants in the low construal level condition increased their shower time ($M_{pre} = 11.24$, $SE_{pre} = 0.63$; $M_{post} = 11.98$, $SE_{post} = 0.81$). Time did not have a significant effect on average shower time ($F(1,142) = 0.54$, $p = .465$, $p\eta^2 = .004$), nor was there an interaction between social distance and time ($F(1,142) = 0.19$, $p = .666$, $p\eta^2 = .001$), nor an interaction between social distance, construal level and time ($F(1,142) = 2.06$, $p = .153$, $p\eta^2 = .014$).

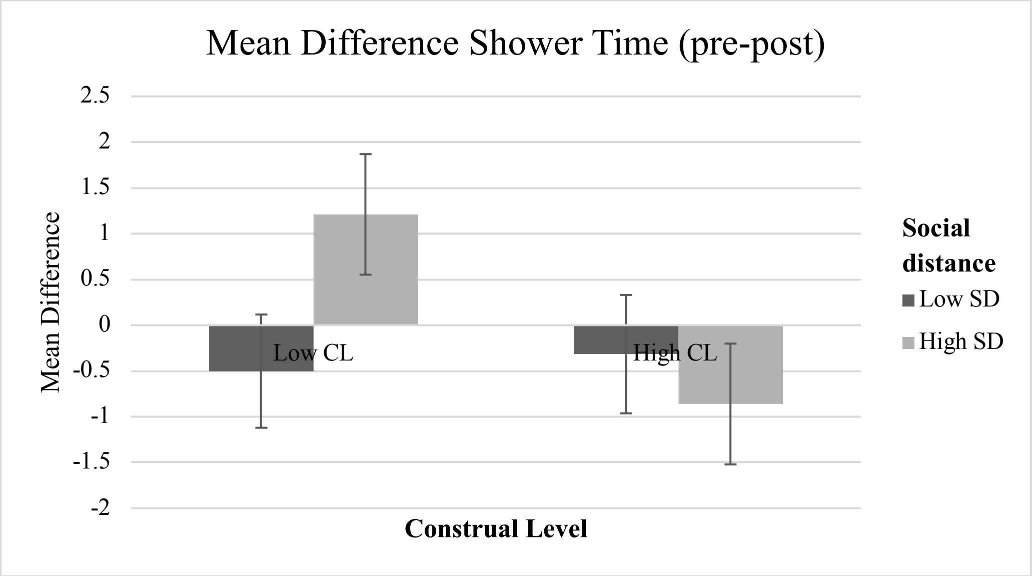

Fig S5.2. Mean difference shower time (post shower time minus pre shower time). Higher scores indicate that participants indicated to shower longer in the post-intervention survey. Error bars represent ± 1 SE.

Self-reported appliance use. The analysis revealed that time did not have a significant effect on appliance use ($F(1,141) = 0.63$, $p = .430$, $p\eta^2 = .004$). There was a significant interaction between time and construal level ($F(1,141) = 4.43$, $p = .037$, $p\eta^2 = .030$), which indicated that participants in the low construal level condition improved on their appliance use ($M_{pre} = 5.12$, $SE_{pre} = 0.11$; $M_{post} = 5.29$, $SE_{post} = 0.10$) and participants in the high construal level condition scored worse on their appliance use in the second survey ($M_{pre} = 5.54$, $SE_{pre} = 0.11$; $M_{post} = 5.43$, $SE_{post} = 0.10$). There was no significant interaction between time and social distance ($F(1,141) = 0.17$, $p = .678$, $p\eta^2 = .001$), or between time, social distance and construal level ($F(1,141) = 2.35$, $p = .128$, $p\eta^2 = .016$).

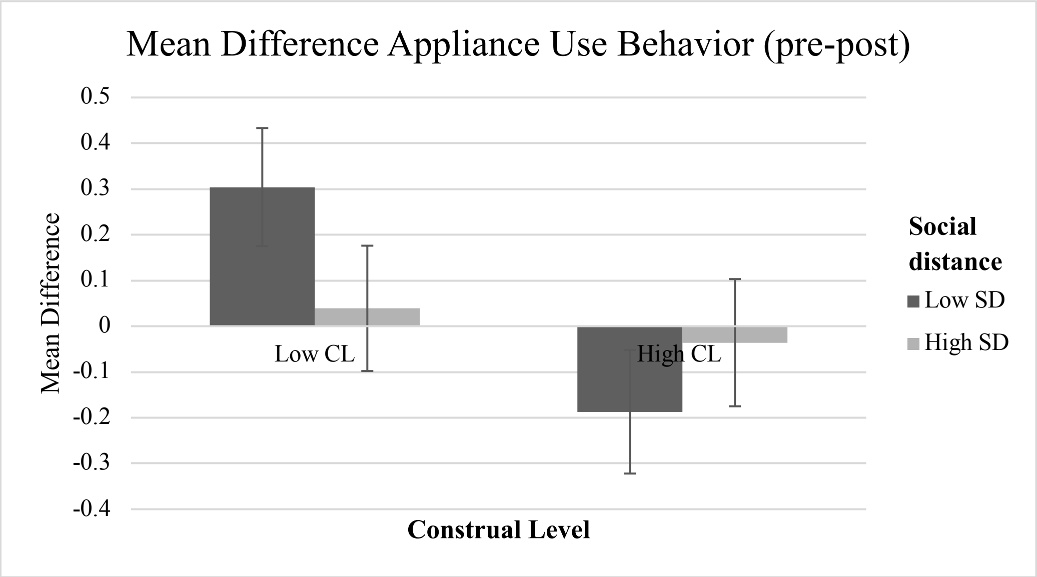

Fig S5.3. Mean difference appliance use (post appliance use behavior minus pre appliance use behavior). Higher scores indicate that participants improved on their appliance use behavior. Error bars represent ± 1 SE.

Self-reported switching off. Repeated measures analysis revealed no significant effect of time on switching off behavior ($F(1,141) = 0.02$, $p = .900$, $p\eta^2 = .000$). Moreover, there were no significant interactions with time: social distance ($F(1,141) = 0.70$, $p = .403$, $p\eta^2 = .005$), construal level ($F(1,141) = 0.18$, $p = .677$, $p\eta^2 = .001$), and the interaction between social distance and construal level ($F(1,141) = 0.01$, $p = .932$, $p\eta^2 = .000$).

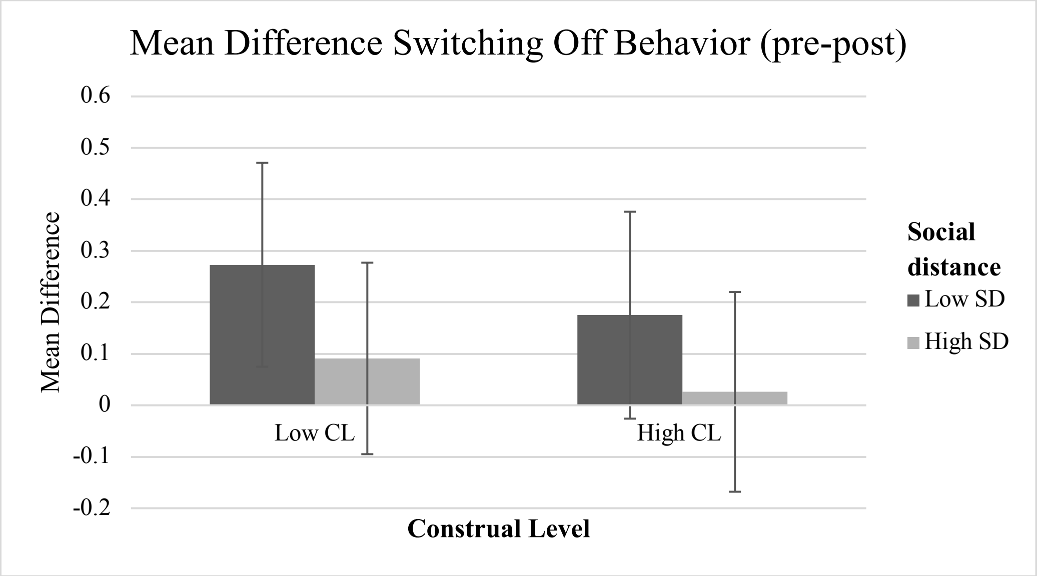

Fig S5.4. Mean difference switching off behavior (post switching off behavior minus pre switching off behavior). Higher scores indicate that participants improved on their switching off behavior. Error bars represent ± 1 SE.

Willingness to pay. Next to the effect on the self-reported energy use behavior, we also asked participants how much they would be willing to pay for the gifts they had received (i.e., either the donation or gift to self). Participants were free to indicate how much they were willing to pay, and after replacing the outliers ($N = 15$) with 2 SDs from the mean, we performed an analysis of variance on willingness to pay. In Figure S5.5, the mean willingness to pay for the gifts can be found. We found a significant effect of social distance, indicating that participants in the high social distance condition were willing to pay more for their gift as compared to participants in the low social distance condition ($F(1,133) = 9.51$, $p = .002$, $p\eta^2 = .067$). Additionally, construal level also had a significant effect on willingness to pay ($F(1,133) = 10.72$, $p = .001$, $p\eta^2 = .075$), showing that participants in the low construal level condition were willing to pay more for their gift than participants in the high construal level condition. Finally, we also found a marginally significant interaction effect between social distance and construal level ($F(1,133) = 3.31$, $p = .071$, $p\eta^2 = .024$), which indicated that participants in the low construal level condition were especially willing to pay more for their

gift when they received the gift to other (i.e., large social distance) as compared to when they received a gift to self (i.e., small social distance).

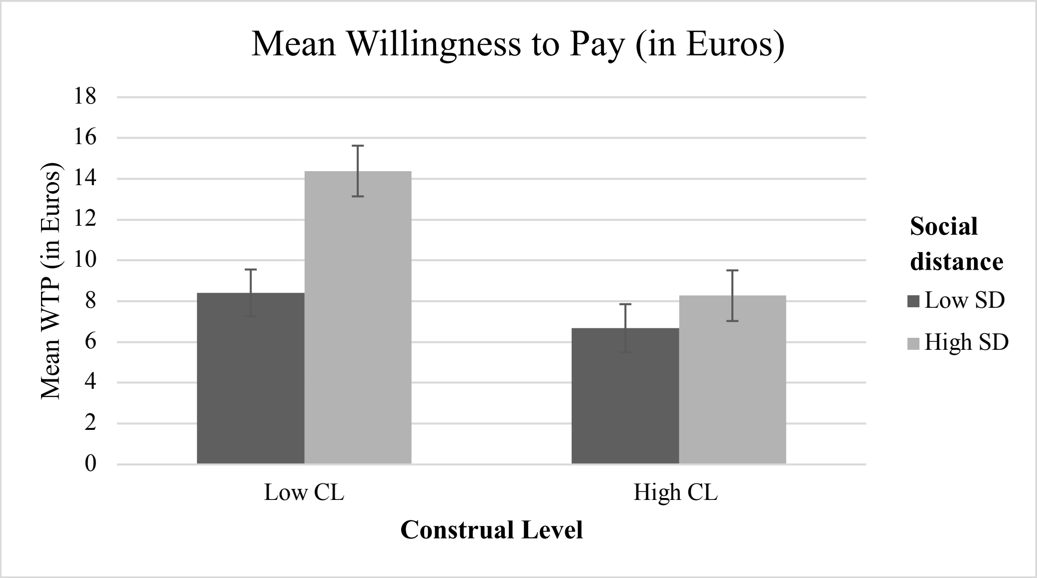

Fig S5.5. Mean willingness to pay for gifts. Higher scores indicate that participants were willing to pay more for the gift. Error bars represent ± 1 SE.

Correlation with water use. As an exploratory analysis, we checked whether participants' willingness to pay for the gift they had received correlated with their water use in week 4. We did, however, not find a significant correlation between willingness to pay and water use in week 4 (Pearson's $r = .059$, $p = .502$).
