# Supplementary material for: Which construal level combinations generate the most effective interventions? A field experiment on energy conservation
Source: PLoS One. 2019 Jan 17;14(1):e0209469. doi: 10.1371/journal.pone.0209469 (PMC6336225; doi:10.1371/journal.pone.0209469)
Supplement: S6 Text — (PDF) [file pone.0209469.s006.pdf]

S6 Text. Survey questions – Not previously published.

Not previously published questions or selected items from published scales

10 items from the Behavior Identification Form (BIF; Vallacher & Wegner, 1989)

1. Washing clothes
2. Cleaning the house
3. Painting a room
4. Paying the rent
5. Caring for houseplants
6. Tooth brushing
7. Taking a test
8. Greeting someone
9. Eating
10. Having a cavity filled

Sustainability of student housing facility

Please state to what extent you agree with each statement below (1 = strongly disagree, 7 = strongly agree)

- *** is a sustainable residence
- *** is concerned about its impact on the environment
- I feel informed about the sustainability efforts of ***

Shower duration and frequency

- How many minutes do you shower for each time you shower (on average)? If you are unsure, please enter your best estimate
- What was the duration of your last shower (in minutes)? If you are unsure, please enter your best estimate
- What do you consider to be a normal shower frequency (per week)? E.g., 'I consider it normal to x times a week'
- When I heat my room at ***, I set the climate control system approximately at (in degrees Celsius)

Pro-environmental behavior

Please state to what extent you agree with the following statements (1 = strongly disagree, 7 = strongly agree)

- I wait until I have a full load before I wash my clothes in the washing machine to save water
- I wash my clothes at a lower temperature to save energy
- I keep the pressure/flow of the shower at a rate lower than what I consider to be ideal to save water
- I limit the time I spend in the shower to reduce my water consumption
- When I'm done charging an appliance, I take the charger out of the socket
- When I boil water, I only boil as much as I need
- When there is nobody in a room I switch off the light
- I keep the shower at a temperature lower than what I consider to be an ideal temperature to save energy
- I turn off the shower when I'm soaping myself down
- I switch appliances off instead of leaving them on standby

How often… (1 = never, 5 = always)

- Are the products you buy organic?
- Do you eat meat?
- Do you bring glass bottles to the recycle bin?
- Do you separate your paper from your waste?
- Do you separate your waste (chemical, plastics, organic)?
- Do you search for environmentally friendly products?
