# Supplementary material for: Which construal level combinations generate the most effective interventions? A field experiment on energy conservation
Source: PLoS One. 2019 Jan 17;14(1):e0209469. doi: 10.1371/journal.pone.0209469 (PMC6336225; doi:10.1371/journal.pone.0209469)
Supplement: S1 Table — (PDF) [file pone.0209469.s007.pdf]

S1 Table. The interaction between the covariates and time in the repeated measures analyses (p-values are reported)

	Water use			Electricity use		Self-report measures			
Interaction with time	Six weeks	Week 0 vs. week 4	Week 0 vs. week 4 (biospheric values as moderator)	Week 0 vs. week 4 (sockets)	Week 0 vs. week 4 (light)	Shower behavior	Shower time	Appliance use	Switching off
Data collection	.667	.864	.858	.057	.595	.844	.435	.969	.415
BIF	.736	.269	.228	.783	**.031**[2]	.098	.413	.797	.867
Biospheric	.200	.360	.476	.124	.110	.893	.090	.879	.560
Age	.136	.916	.870	.260	.598	**.037**[3]	.268	.838	.437
Gender	.984	.558	.810	**.004**[1]	.063	.371	**.015**[4]	.204	.867

Note. Significant interactions between one of the covariates and time are depicted in bold.[1] The significant interaction between time and gender indicated that men did not change their socket use, whereas women increased their socket use. [2]The interaction between BIF and time indicated that people who scored higher on BIF used more light in week 4 as compared to the week before the intervention (B = 4.81). [3]The interaction between age and time indicated that older participants reduced their shower behavior more than younger participants (B = 0.05). [4]The interaction between gender and time indicated that men reduced their shower time (M_{pre} = 10.98, SE_{pre} = 0.67; M_{post} = 10.03, SE_{post} = 0.85), whereas women increased their shower time (M_{pre} = 11.01, SE_{pre} = 0.65; M_{post} = 11.76, SE_{post} = 0.82).
