# Supplementary material for: Which construal level combinations generate the most effective interventions? A field experiment on energy conservation
Source: PLoS One. 2019 Jan 17;14(1):e0209469. doi: 10.1371/journal.pone.0209469 (PMC6336225; doi:10.1371/journal.pone.0209469)
Supplement: S2 Table — (PDF) [file pone.0209469.s008.pdf]

S2 Table. Main effects in repeated measures analyses on average energy and water use.

	Water (6 week period)				Water (week 0 vs week 4)				Sockets (week 0 vs week 4)				Lights (week 0 vs week 4)			
	df	*F*	*p*	$p\eta^2$	*df*	*F*	*p*	$p\eta^2$	*df*	*F*	*p*	$p\eta^2$	*df*	*F*	*p*	$p\eta^2$
Intercept		4.15	.043	.027		3.42	.066	.022		3.04	.084	.023		5.77	.018	.042
Wave		0.16	.690	.001		0.10	.754	.001		0.28	.601	.002		2.74	.101	.021
Biospheric		2.71	.102	.018		1.07	.303	.007		0.28	.599	.002		0.01	.930	.000
BIF		0.08	.784	.001		0.22	.644	.001		2.76	.099	.021		2.03	.157	.015
Age		1.69	.195	.011		2.28	.134	.015		0.63	.431	.005		2.34	.128	.018
Gender		0.45	.502	.003		0.36	.552	.002		1.96	.164	.015		0.01	.941	.000
Social distance		0.39	.532	.003		0.12	.734	.001		1.04	.310	.008		0.00	.999	.000
CL		0.22	.644	.001		0.14	.710	.001		2.98	.087	.022		0.55	.460	.004
Social distance*CL		1.01	.317	.007		0.73	.396	.005		0.30	.588	.002		0.71	.401	.005
Error	148				150				130				130			

Note. CL = Construal level. The average use for the six week period as a dependent measure is shown in the first column and the average use of week 0 and week 4 is shown as the dependent measure in the latter three columns.
