# Supplementary material for: Which construal level combinations generate the most effective interventions? A field experiment on energy conservation
Source: PLoS One. 2019 Jan 17;14(1):e0209469. doi: 10.1371/journal.pone.0209469 (PMC6336225; doi:10.1371/journal.pone.0209469)
Supplement: S3 Table — (PDF) [file pone.0209469.s009.pdf]

S3 Table. Main effects in repeated measures analyses on the average self-report measures.

	Shower behavior				Shower time				Appliance use				Switching off			
	df	*F*	*p*	*p*η^2	*df*	*F*	*p*	*p*η^2	*df*	*F*	*p*	*p*η^2	*df*	*F*	*p*	*p*η^2
Intercept		0.890	.346	.006		8.45	.004	.056		6.77	.010	.046		1.10	.297	.008
Wave		4.380	.038	.030		3.49	.064	.024		2.61	.108	.018		2.23	.137	.016
Biospheric		9.210	.003	.061		0.37	.546	.003		23.32	.000	.142		19.56	.000	.122
BIF		0.170	.683	.001		0.73	.396	.005		2.18	.142	.015		0.05	.818	.000
Age		15.980	.000	.102		0.17	.685	.001		6.51	.012	.044		11.39	.001	.075
Gender		0.145	.704	.001		0.41	.523	.003		4.12	.044	.028		0.40	.526	.003
Social distance		0.182	.670	.001		1.13	.291	.008		1.83	.178	.013		0.001	.976	.000
CLT		1.960	.163	.014		1.75	.188	.012		4.27	.041	.029		1.63	.204	.011
Social distance*CLT		0.360	.553	.003		0.83	.364	.006		3.05	.083	.021		1.64	.203	.011
Error	141				142				141				141			

Note. Average of pre- and post-intervention survey scores taken as dependent measure.
