# Supplementary material for: Which construal level combinations generate the most effective interventions? A field experiment on energy conservation
Source: PLoS One. 2019 Jan 17;14(1):e0209469. doi: 10.1371/journal.pone.0209469 (PMC6336225; doi:10.1371/journal.pone.0209469)
Supplement: S4 Table — (PDF) [file pone.0209469.s010.pdf]

S4 Table. Correlations between self-reported values and measures warm water and electricity use during baseline.

	Water	Sockets	Light	Shower time	Shower behavior	Appliance use	Switching off	Hedonic	Egoistic	Altruistic	Biospheric	Efficacy (2 items)	Env. identity
Sockets	.137*	-											
Light	.191**	.375***	-										
Shower time	.354***	-.031	-.015	-									
Shower behavior	-.142**	-.045	.054	-.283***	-								
Appliance use	-.064	-.212***	-.139*	-.229***	.441***	-							
Switching off	-.074	-.215***	-.174**	-.119*	.360***	.453***	-						
Hedonic	-.026	.009	.008	.003	-.159**	-.051	-.038	-					
Egoistic	.004	.070	-.002	.025	-.118*	-.087	.005	.434***	-				
Altruistic	-.061	.070	.032	-.115	.102	.114	.142**	.314***	.208***	-			
Biospheric	-.011	.028	-.036	-.074	.271***	.359***	.308***	.268***	.145**	.704***	-		
Efficacy (2 items)	-.015	.035	.036	-.026	.241***	.287***	.247***	.029	.043	.256***	.250***	-	
Environmental identity	-.102	-.060	-.156**	-.159**	.396***	.459***	.386***	-.116	-.040	.126*	.437***	.235***	-
BIF	.044	.212***	.068	.068	.131*	.186***	.110	-.046	.006	.081	.128*	.156**	.152**

Note. * $p < .10$, ** $p < .05$, *** $p < .01$
