# Supplementary material for: Which construal level combinations generate the most effective interventions? A field experiment on energy conservation
Source: PLoS One. 2019 Jan 17;14(1):e0209469. doi: 10.1371/journal.pone.0209469 (PMC6336225; doi:10.1371/journal.pone.0209469)
Supplement: S5 Table — (PDF) [file pone.0209469.s011.pdf]

S5 Table. The effect of time and the interaction between time and the independent variables in the repeated measures analysis on objectively measured water use

	Baseline vs. Week 1				Baseline vs. Week 2				Baseline vs. Week 3				Baseline vs. Week 5			
	df	*F*	*p*	*p*η²	*df*	*F*	*p*	*p*η²	*df*	*F*	*p*	*p*η²	*df*	*F*	*p*	*p*η²
Time		1.17	.280	.007		0.50	.479	.003		0.19	.668	.001		3.89	**.050**[1]	.026
Time*Wave		1.98	.161	.012		0.01	.930	.000		0.07	.786	.000		0.04	.834	.000
Time*Biospheric		0.72	.398	.005		1.10	.295	.007		2.19	.141	.014		5.25	**.023**[2]	.034
Time*BIF		0.03	.858	.000		0.17	.677	.001		0.12	.734	.001		0.09	.771	.001
Time*Age		1.88	.173	.012		3.49	.064	.022		0.42	.520	.003		0.73	.396	.005
Time*Gender		0.05	.823	.000		0.56	.455	.004		0.01	.936	.000		0.22	.642	.001
Time*Social distance		0.30	.586	.002		0.31	.577	.002		0.18	.670	.001		0.01	.920	.000
Time*CLT		0.003	.955	.000		0.47	.493	.003		0.49	.486	.003		0.45	.504	.003
Time*Social distance*CLT		0.36	.547	.002		1.21	.273	.008		2.37	.126	.015		0.63	.429	.004
Error	158				152				152				148			

Note. Significant effects are depicted in bold. [1]The significant interaction of time in week 5 indicated that (compared to the control group) all participants used less water as compared to the baseline period. [2]The interaction between biospheric values and time indicated that the higher people scored on biospheric values the more water they used in week 5 as compared to baseline (*B* = 2.69).
