# Supplementary figures and images for: Which construal level combinations generate the most effective interventions? A field experiment on energy conservation
Source: PLoS One. 2019 Jan 17;14(1):e0209469. doi: 10.1371/journal.pone.0209469 (PMC6336225; doi:10.1371/journal.pone.0209469)

**S1 Fig. Absolute water use throughout 6-week intervention period.**

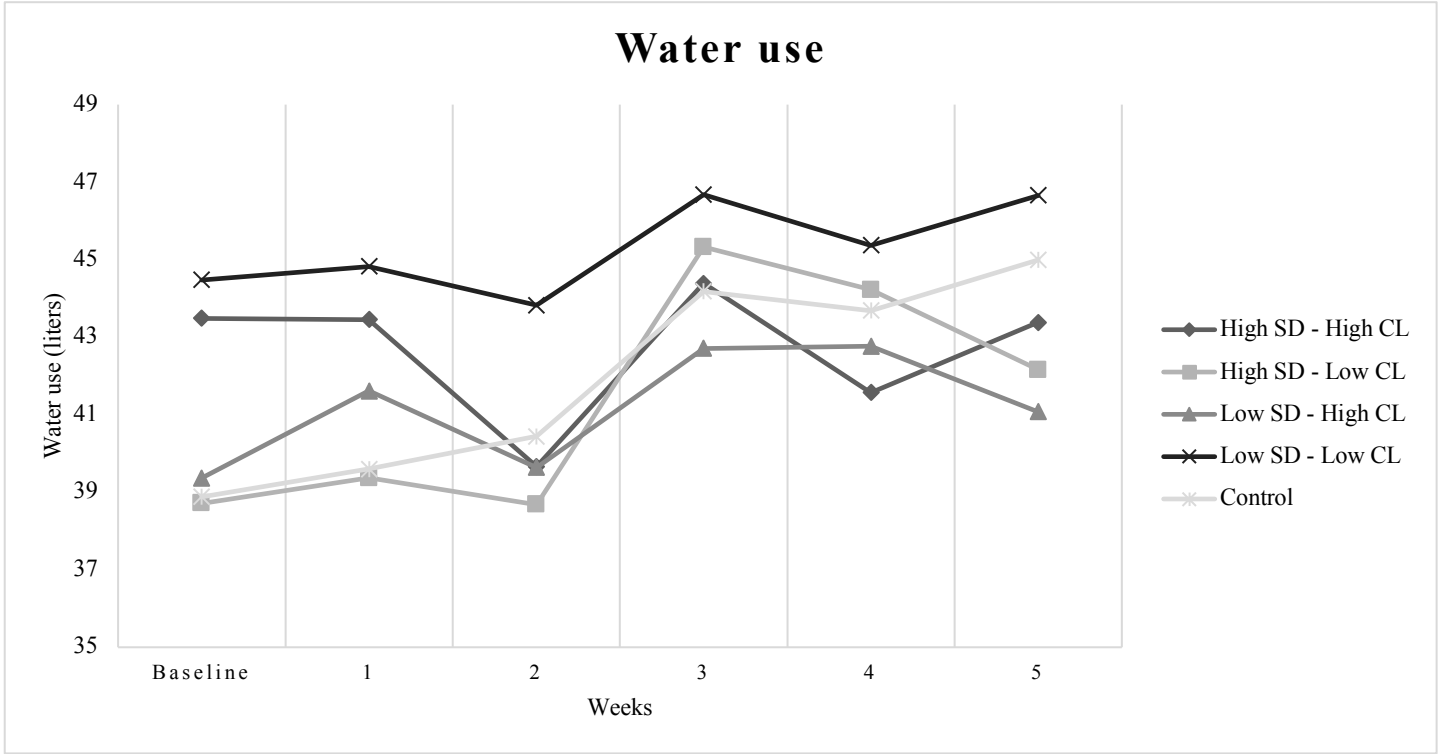

Supplement: S1 Fig — (PDF) [file pone.0209469.s012.pdf]
